# Magnetic Nanoparticles in Precision Cancer Therapy: From Synthesis and Biological Interactions to Clinical Translation

**DOI:** 10.3390/ijms27219938

**Published:** 2026-11-06

**Authors:** Jing Wang, Shizhi Xu, Seyedeh Maryam Hosseinikhah, Wei Zhang, Xiao Zhang, Andrea Sacchetti

**Affiliations:** 1 Department of Pathology, Erasmus University Medical Center, 3015 GD Rotterdam, The Netherlands; j.wang@erasmusmc.nl (J.W.); s.hosseinikhah@erasmusmc.nl (S.M.H.); 2 Institute of Frontier Medical Sciences, Jilin University, Changchun 130021, China; szxu20@mails.jlu.edu.cn; 3 Department of Clinical Genetics, Erasmus University Medical Center, Sophia Children’s Hospital, 3015 GD Rotterdam, The Netherlands; w.zhang@erasmusmc.nl; 4 State Key Laboratory for Diagnosis and Treatment of Severe Zoonotic Infectious Diseases, Key Laboratory for Zoonosis Research of the Ministry of Education, Institute of Zoonosis, College of Veterinary Medicine, Jilin University, Changchun 130062, China; zxiao21@mails.jlu.edu.cn

**Keywords:** magnetic nanoparticles, hyperthermia, tumor microenvironment, nanorobots

## Abstract

Cancer treatment increasingly relies on integrated therapeutic approaches, particularly for solid tumors that remain difficult to eradicate using a single modality. Magnetic nanoparticles (MNPs) have emerged as versatile therapeutic platforms owing to their magnetic responsiveness and tunable physicochemical properties. Advances in synthesis methods enable precise control over MNP size, morphology, composition, and surface chemistry, shaping their biological behavior across multiple scales. At the cellular level, these properties regulate nanoparticle uptake, intracellular trafficking, and subcellular interactions. At the tissue and systemic levels, administration route, vascular transport, magnetic targeting, and ligand-mediated recognition influence biodistribution, tumor accumulation, and cellular selectivity. Material composition and surface functionalization further determine responsiveness to external or endogenous stimuli and mediate therapeutic effects, including heat generation, controlled drug release, catalytic activity, and reactive oxygen species production. These features allow MNP-based systems to support localized hyperthermia, drug delivery, catalytic therapies, and embolization-based interventions, making them attractive for treating deep-seated and inoperable tumors. However, heterogeneous tumor delivery, variable activation efficiency, off-target toxicity, and long-term safety concerns remain major barriers to clinical translation. Overall, this review summarizes recent advances and current limitations in MNP-based cancer therapy, providing a multiscale perspective that links material design, biological interactions, systemic delivery, therapeutic activation, and translational challenges.

## 1. Introduction

The incidence of cancer is progressively increasing over the years, accompanied by a significant health burden [1]. According to the global cancer statistics report published in 2024, approximately 20.0 million new cancer cases and 9.7 million cancer-related deaths occurred worldwide, and the annual number of new cases is projected to reach 35 million by 2050 [2]. Surgical resection is mostly effective in early-stage cancers [3]. However, surgical treatment remains challenging and may not achieve complete (R0) resection for cancers located in anatomically inaccessible regions or that have progressed to advanced stages and metastasized to distant organs. Patients with brain, lung, or other distant metastases have a poor prognosis, and effective therapy options remain limited [4]. For patients with advanced cancer, chemotherapy regimens and immunotherapy are still the main therapeutic approaches [5]. However, conventional chemotherapy is limited by non-selective systemic distribution and dose-limiting toxicity. Even the newer generation of chemotherapy does not significantly improve the long-term survival rate of such patients, since drug toxicity and resistance, months to years after regimen initiation, are often unavoidable [6]. Although immunotherapy has a higher long-term success rate, it is only applicable to selected tumor types and is limited by heterogeneous responses, an immunosuppressive TME, and primary or acquired resistance, and it is still associated with a high risk of serious adverse effects [7]. Although molecular therapies targeting PARP [8], BRAF [9], HER2 [10], etc., have improved treatment specificity, their clinical efficacy may be limited by tumor heterogeneity, resistance, and treatment-related toxicity. Radiotherapy also has major limitations, since it can cause collateral damage to surrounding normal tissues and is less effective in hypoxic or radioresistant tumors [11]. Moreover, in aggressive or advanced cancer, the probability of microscopic metastases falling outside the radiation field may be high. These limitations underscore the critical need for alternative targeted treatment modalities.

Nanomaterials, used alone or in combination with other therapies, might be one of these options. Various nanomaterials have been explored for biomedical applications, including lipid nanoparticles, polymeric nanoparticles, dendrimers, and extracellular vesicles. Among these, magnetic nanoparticles (MNPs) stand out as a particularly promising platform for cancer therapy due to their unique responsiveness to external magnetic fields and highly tunable physicochemical properties. MNPs can be remotely guided to tumor sites and convert electromagnetic energy from external magnetic fields into localized heat, enabling selective tumor ablation or controlled drug release within deep-seated tumors [12]. Among different types of MNPs, iron oxide-based MNPs have good biocompatibility and can be metabolized through endogenous iron pathways. Due to these properties, certain nano-iron oxide formulations have been approved by the U.S. Food and Drug Administration (FDA) for clinical use in diagnostic imaging and other medical applications. Their compatibility with imaging systems enables real-time tracking and image-guided therapy [13].

In this review, we provide an overview of the potential applications of MNPs in cancer therapy. We compare their fundamental design and physicochemical properties and move through the latest research progress in their biological interactions at the cellular and tumor microenvironment (TME) levels. Unlike recent reviews focusing on individual MNP applications [14], materials, or tumor types, this review links MNP physicochemical properties and biological interactions to therapeutic performance. It also covers less-reviewed applications, including immunoseparation, magnetic embolization, and nanocatalytic therapy, while highlighting the major barriers that still hinder clinical translation to cancer therapy.

## 2. Design at the Nanoscale: Structure, Preparation Methods, and Properties of MNPs

MNPs exhibit unique physicochemical properties, particularly superparamagnetism, high surface-to-volume ratio, and modifiable surface hydrophilicity and lipophilicity [15]. Current research mainly focuses on iron oxide-based systems, especially Fe_3_O_4_ and γ-Fe_2_O_3_, because they are magnetic, readily available, and among the most abundant trace elements in the human body, thus exhibiting good biocompatibility. These systems have been developed for serving both as contrast agents in magnetic resonance imaging (MRI) and as heat mediators in cancer therapy [16].

Despite the achievements that have been made in fundamental research, several challenges remain at the material-design step. The nanoparticle design principle “one-size- or one-shape-fits-all purposes” often faces some limits, including non-optimal heating efficiency. The heating efficiency is normally quantified by specific loss power (SLP) [17], using a ratio related to the energy generated by MNPs and the external magnetic field provided. When the MNPs have a low heating efficiency, high doses are necessary to achieve therapeutic efficacy. Design with insufficient control over size distribution and morphological anisotropy during synthesis also often leads to heterogeneous magnetic responses both in vitro and in vivo, and an increased tendency toward aggregation. These two elements also pose a major challenge for large-scale manufacturing.

These problems in therapeutic performance fundamentally originate from material-level physical limitations. Rational design at the synthesis stage, such as introducing shape-controlled architectures or elemental doping, offers a bottom-up strategy to enhance magnetic anisotropy, thereby improving energy conversion efficiency [18]. In this section, we will examine how material-level optimization, including synthesis strategies, structural engineering, and physicochemical properties, can influence the metabolic fate and biomedical performance of MNPs.

### 2.1. Structure of MNPs

**Figure 1:**
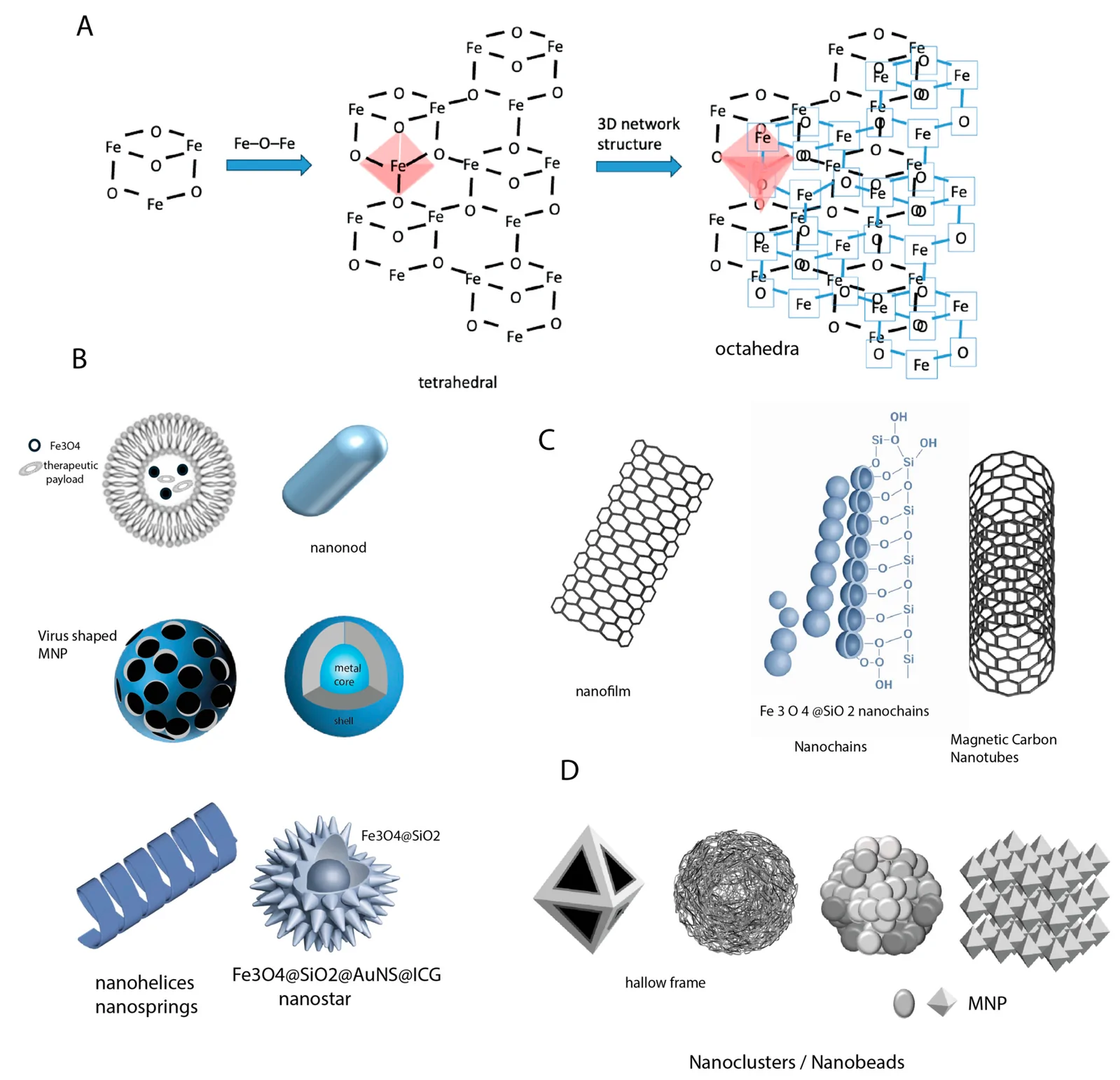
Representative structures of MNPs. (**A**) Structure of iron oxides. (**B**) Representative structures of single MNPs in 0D, including core–shell, rod-like, and virus-like structures. (**C**) 1D and 2D nanostructures, including nanofilms, nanochains, and nanotubes. (**D**) 3D structures of MNPs, including nanostars, nanoframes, and nanoclusters.

MNPs are inorganic zero-dimensional (0D) materials with metal-based structures. The center of magnetic nano-ions, the magnetic core, is generally composed of iron and cobalt alloys (such as CoPt_3_, FePt, FeZn, etc.) or iron oxides, including magnetite (Fe_3_O_4_) and maghemite (γ-Fe_2_O_3_) [19]. Fe_3_O_4_ has an inverse spinel structure, in which oxygen anions are arranged in a face-centered cubic (fcc) frame, while iron cations occupy both tetrahedral and octahedral interstitial sites. γ-Fe_2_O_3_ exhibits a similar structure but contains cation vacancies in the octahedral sites to maintain a neutral charge (Figure 1A). Maghemite is often formed when iron oxides are added to the chosen metal core, which then becomes magnetic, forming an M-Fe_2_O_4_ structure, where M is a +2 cation such as Mn, Fe, Co, or Ni [20]. MNPs can also be based on a mix of compounds normally chosen from copper, zinc, strontium, and barium [19]. This doped ferrite structure can provide adjustable magnetic responses. Differences in crystal structure and cation distribution between Fe_3_O_4_ and γ-Fe_2_O_3_ can significantly influence their magnetic behavior and stability.

Moreover, synthesis conditions and nanoparticle design can be adjusted to generate MNPs with diverse morphologies. Particle shape strongly influences the physicochemical properties of MNPs, including magnetic anisotropy and surface-to-volume ratio, which in turn affect chemical reactivity, colloidal stability, and energy conversion efficiency. Spherical nanoparticles are the most commonly prepared ones, because of their relatively simple synthesis and high reproducibility. However, their high symmetry is generally associated with lower magnetic anisotropy [21]. In contrast, nanocubes [22] and octahedral nanoparticles [23] often exhibit enhanced magnetic anisotropy and improved heating performance under alternating magnetic fields (AMFs). One-dimensional nanostructures with a relatively linear shape, such as nanorods and nanowires, possess greater shape anisotropy, which can further strengthen magnetic responses and promote directional motion [24].

More complex morphologies, including nanoclovers [25] and nanostars [26], display asymmetric architectures that influence magnetic torque and dynamic behaviors such as rotation, oscillation, and reorientation. However, their intricate structures may also increase the tendency to aggregate in biological environments, potentially affecting vascular transport, biodistribution, and biological activity (Figure 1B).

To improve MNP stability and biocompatibility, surface modification is applied. Surface modification normally appears as coating layers forming core–shell structures, which protect the magnetic core from oxidation and decay while also reducing particle aggregation through electrostatic or steric repulsion. The coating layers can be made of different functional materials. Inorganic coatings such as silica can provide permeable structures suitable for drug loading, while gold or silver shells may improve chemical stability by preventing undesired reactions in biological environments and introduce additional functionalities like the plasmonic effect [27]. In this effect, MNPs respond to electromagnetic waves through synchronous vibrations of their surface electrons, leading to enhanced light absorption and the conversion of light into heat. In addition, surface modulation with ligands such as antibodies, peptides, or folic acid enables MNPs to target specific cells. Antibodies may be immunogenic and costly, whereas peptides are smaller and penetrate tissues more readily but are susceptible to enzymatic degradation. Aptamers [28], i.e., short DNA or RNA molecules that can bind to selective targets, offer the advantage of low immunogenicity and easy modification, although their stability may be limited by nucleases [29]. More complex systems have also been developed by combining magnetic materials with biological components. For example, magnetic liposomes have been produced, in which lipid bilayers encapsulate MNPs, providing an external surface that can enhance cellular uptake and enable biosensing applications using lipophilic fluorescent probes [30]. Magnetic micelles formed from amphiphilic polymers can incorporate magnetic cores and hydrophobic drugs at the same time. Janus nanoparticles, which consist of two distinct domains, allow us to combine different functionalities within a single structure [31]. MNP conjugation with biomolecules such as DNA, RNA, or proteins can enable the development of delivery systems for these molecules and can be used for genetic or antigen-based therapies. Similarly, drugs can also be loaded onto or within magnetic carriers to be specifically targeted to tumors. Magnetic-field-assisted localization and controlled release at the tumor site [32,33] are important factors in determining whether MNP-based drug delivery will be successful or not.

The size of MNPs is typically controlled within the 1–100 nm range, as particle size critically influences both magnetic behavior and biological performance. When their diameter decreases to approximately 10–20 nm, MNPs can exhibit superparamagnetic properties while maintaining favorable biodistribution profiles. Beyond the characteristics of individual nanoparticles, the assembly of multiple MNPs into higher-order structures offers additional opportunities to overcome some limitations of single-core systems, including relatively low magnetic responsiveness, while modulating SLP and surface area. Among these architectures, magnetic nanochains consist of linearly arranged nanoparticles and can reach lengths on the micrometer scale [34]. Compared with other multicore structures, nanochains may provide enhanced magnetic guidance and improved penetration through biological environments. For example, Fe_3_O_4_@SiO_2_ nanochains exhibit reduced magnetic coupling between adjacent particles, resulting in stronger local magnetic fields [35]. Such assembled structures have been explored for biomarker capture, drug delivery, and magnetic hyperthermia applications [36]. Despite these advantages, one-dimensional architectures may present several limitations for solid tumor treatment. Their synthesis is generally more complex, which may affect drug-loading stability and lead to less predictable release kinetics. In physiological environments, these structures can also undergo partial structural degradation, potentially compromising their functionality. Furthermore, compared with zero-dimensional single-core nanoparticles, elongated or tubular nanostructures may face greater steric and physical resistance when navigating the extracellular matrix, crossing biological barriers, or interacting with cellular membranes. Since many deep-seated or inoperable tumors are already difficult to access, these orientation-dependent interactions may contribute to non-uniform intratumoral distribution and reduced cellular uptake.

Two-dimensional (2D) nanofilms possess a large and continuous surface area, making them attractive platforms for biosensing and surface functionalization. Their planar architecture provides a high-density interface for the immobilization of ligands, such as antibodies and DNA probes, thereby enhancing target recognition and molecular interactions. However, these same surface characteristics can also promote nonspecific adsorption of biomolecules in vivo, leading to protein corona formation, altered colloidal stability, and reduced circulation performance. Consequently, their application in systemic drug delivery remains limited. As a result, 2D nanofilms are more commonly employed in in vitro diagnostic applications than in in vivo drug delivery systems (Figure 1C).

2D nanofilms can be further assembled into 3D structures through cross-linking or assembly strategies, such as ligand-mediated assembly, electrostatic interactions, or magnetic field-induced methods. Unlike the lower-dimensional systems, these 3D magnetic materials normally do not enter the systematic blood flow but remain in the same place, providing enhanced structural complexity and mechanical support, and enabling applications that require spatial organization and bulk functionality. For example, 3D magnetic materials have been explored in bone tissue engineering, where permeable magnetic scaffolds, such as Fe_3_O_4_-based bioactive glass-related composites, were applied to support cell proliferation and bone regeneration under magnetic stimulation [37]. Other 3D architectures such as multilayer structures have better interface control. Among these structures, hollow magnetic nanoframes [38], formed through selective etching of nanocubes, can provide several internal cavities that can be used as storage units, increasing the drug-loading capacity. Additionally, MNPs can also be used as cross-linking nodes in polymeric matrices. This can enhance mechanical strength while providing functional sites for further modification [39]. However, compared with zero-dimensional (0D) MNPs, three-dimensional (3D) architectures are more difficult to fabricate in a reproducible and scalable manner. Moreover, non-uniform MNP distribution and aggregation within these structures may generate localized heating hotspots under AMFs, potentially leading to uneven therapeutic effects and unintended damage to adjacent healthy tissues (Figure 1D).

Overall, these structural designs and surface modification strategies allow MNPs to serve as multifunctional platforms for applications in diagnosis, therapy, and monitoring of solid cancers.

### 2.2. Preparation Methods of MNPs

Several iron oxide-based core synthesis methods have been approved for clinical applications [40]. MNP preparation procedures include chemical, physical and biological methods, which are summarized in Table 1. The main goal of all the preparation methods is to achieve better control over size, shape, and crystallinity of MNPs. Particle morphology is determined by the balance among nucleation, crystal growth, surface-energy minimization, and facet-selective stabilization during synthesis [41]. The synthesis parameters are the core factors determining these processes. During synthesis, particle size can be precisely controlled by adjusting pH, salt concentration, alkali strength, reaction temperature [42], or the properties of the magnetosome membrane vesicles that synthesize MNPs [43].

By changing the proportion of surfactants (such as oleic, dodecanoic, and octanoic acid [44]) or precursors, particle crystals can grow along specific plane orientations, thereby controlling morphology and anisotropy. Coprecipitation commonly produces spherical particles because rapid nucleation and isotropic growth favor the minimization of surface energy, whereas thermal decomposition [45], hydrothermal, and solvothermal methods provide greater control over reaction kinetics and crystal-facet growth, enabling the formation of spherical, cubic, rod-like, and flower-like structures. In particular, selective binding of surfactants to specific crystal facets can promote anisotropic growth and generate nanocubes [46] or nanorods, while oriented aggregation or fusion of primary nanocrystals can produce multicore and nanoflower structures [47]. Microemulsion synthesis generally yields small spherical particles [48] because the nanodroplet reactors spatially restrict particle growth, whereas the less-controlled reaction conditions of combustion and green synthesis often result in broader or irregular morphologies [49]. Physical methods such as laser ablation and arc discharge primarily produce spherical particles through the rapid cooling and condensation of vaporized materials, although their size distributions may be relatively broad. The synthesis method can also directly influence the subsequent heat-generation performance of MNPs by controlling their composition and magnetic properties. For example, sol–gel-derived (Mn_1−x_Zn_x_)Fe_2_O_4_ nanoparticles have been compositionally tuned by varying the Mn/Zn ratio for magnetocaloric applications, illustrating how synthesis-level compositional control can be exploited to optimize magnetic heating behavior [50].

Chemical methods rely on chemical reactions to convert precursors into MNPs and are generally cost-effective, offer good control over particle size and morphology, and are suitable for large-scale production. In recent years, continuous-flow synthesis has emerged as an attractive alternative to conventional batch processes, which are often limited by inter-batch variability and limited scalability. The use of micro- and millimeter-scale reactors enables tighter control of reaction parameters, while improving heat and mass transfer throughout the system. As a result, continuous-flow approaches offer promising opportunities for the scalable and reproducible production of superparamagnetic iron oxide nanoparticles (SPIONs). However, the downside of many chemical synthesis routes is that they still depend on reagents and solvents that raise environmental concerns. In contrast, biological synthesis methods exploit enzymatic activity and cellular metabolism to generate MNPs under more cost-effective and environmentally sustainable conditions. Green synthesis strategies based on plant extracts or microorganisms can reduce the use of hazardous chemicals, offering an eco-friendly and cost-effective alternative to conventional synthesis methods [51]. However, these biological methods generally exhibit lower control over particle size, morphology, and composition compared with physical methods, which often yield materials with cleaner ligand-free surfaces (Table 1).

In the process towards actual use, post-synthetic functionalization and hybrid integration are commonly used to modify MNPs and enable their multifunctionality [52]. All of these need to comply with one premise: maintaining, at the same time, both the activity of the ligand and good physicochemical properties. MNPs can also be modified and selected with the help of microfluidic systems [53] and high-performance liquid chromatography (HPLC) [54]. Methods like chemical vapor deposition are used for film structures [55]. Manufacturing models like anatomical phantoms are also used to predict the clinical response of MNPs with defined architectures [56].

Future research on MNP synthesis methods is expected to focus on automated control, environmentally friendly procedures, better reproducibility, and the development of multifunctional hybrid architectures [57]. The integration with artificial intelligence (AI) and machine learning approaches to optimize the synthesis processes may enable us to predict the optimal combination of synthesis parameters needed, such as pH, temperature, and precursor concentration, and define the properties of the final particles, including size and distribution, improving the precision and reproducibility of nanoparticle fabrication. Taken together, these approaches may help set up protocols for MNP preparation with improved efficiency, cost-effectiveness, controllability, and safety.

**Table 1:** Overview and comparison of commonly used synthesis methods for MNPs.

	Preparation Methods	Advantages	Disadvantages	References
Predominant Chemical Synthesis Methods	Solution combustion method: rapid synthesis of nanomaterials via ignition of a solution containing metal precursors and fuel, driven by an exothermic redox reaction	Products with high specific surface area and high reactivity. Raw materials are cheap. Simple process with high yield Low-cost and potentially scalable.	Poor controllability of combustion process Coarse product particle diameter. Limited reproducibility and GMP compatibility owing to difficult temperature and emission control.	[58,59]
	Microemulsion method: uses oil–water interface nanoreactors	Good particle crystallinity. Effectively controlled product size, shape and composition Low synthesis temperature. Good laboratory-scale reproducibility.	Large molecular gap. Low yield, high solvent and surfactant consumption, difficult purification, and poor scalability and GMP compatibility.	[60,61,62]
	Sol–gel method: precursor solution forms a sol, which undergoes hydrolysis and condensation to yield a 3D gel, followed by heat treatment to obtain crystalline products	High product purity and uniformity, Mild processing conditions (atmospheric pressure and low temperature) Precise control of nanoparticles composition, size, etc.	Longer reaction time. Drying, calcination, and residual-solvent control increase cost and complicate GMP production.	[63,64,65,66]
	Solvothermal synthesis method: synthesis in a sealed vessel under high temperature and pressure, where precursors react in a non-aqueous solvent to form nanocrystals	Simple operation, non-toxic Size, shape distribution and crystallinity of MNPs can be precisely controlled.	Reaction requires high pressure. Difficult to mass-produce.	[67,68,69,70]
	Hydrothermal synthesis: synthesis in aqueous solution under high temperature and pressure via hydrolysis and oxidation reactions	High purity, good crystallinity, uniform composition, and good aqueous dispersibility suitable for biomedical applications.	Requires high temperature and pressure, with limited control over ultrasmall particle size, slow reaction kinetics, scalability challenges, and potential purity limitations.	[71,72]
	Coprecipitation method: mixing metal salt solutions (e.g., Fe^2+^/Fe^3+^) with a base under inert atmosphere to form precipitated nanoparticles	Simple preparation process. Low cost. Preparation conditions are easy to control. Short synthesis cycle.	Lack of control over particle shape and size. Batch reproducibility is sensitive to pH, mixing, temperature, and oxidation.	[73,74]
	Thermal decomposition: heating metal–organic precursors in high-boiling organic solvents with surfactants to form nanoparticles	High crystalline, monodisperse nanoparticles with excellent size and shape control.	Hydrophobic products requiring post-functionalization, along with the use of toxic organic solvents, high energy consumption, complex purification, and limited dispersibility in aqueous media.	[75,76]
	Polyol Process: reflux synthesis using liquid polyols as solvent, reducing agent, and stabilizer to prevent aggregation	One-step synthesis of highly crystalline hydrophilic MNPs ranging from single-core spheres to intricate nanoflowers.	Long reaction time, use of toxic solvents, and challenges in separating products from the solvent.	[77]
Physical and Emerging Synthesis Methods	Laser Ablation in Liquids: nanoparticles are formed by irradiating a metal target in liquid with a high-energy laser, causing the material to evaporate and then condense into nanoscale particles	Simple and contamination-free process, producing high-purity nanoparticles without stabilizers or costly chemicals, with rapid and environmentally friendly synthesis.	Poor control over particle size distribution and crystal structure, along with complex equipment setup and high energy consumption.	[78,79]
	Arc Discharge: vaporized material condenses into nanoparticles in the gas phase around high-temperature plasma regions	Clean, waste-free process producing nanoparticles with excellent thermal and magnetic properties.	Low industrial scalability and efficiency, with challenges in controlling particle size and thickness.	[80,81]
Biological and Green Synthesis	Plant-mediated Synthesis: use plant extracts (e.g., leaves, bark, fruits) as reducing and stabilizing agents	Low cost and naturally biocompatible surface functionalization. Potentially scalable under standardized conditions.	Limited control over particle size and magnetic properties, with significant batch-to-batch variability.	[82,83,84]
	Microbial Synthesis: microorganisms (e.g., magnetotactic bacteria, fungi, algae) produce MNPs via biologically mediated reduction, including the formation of magnetosomes	Produces highly uniform morphologies (e.g., chain-like magnetosomes) with excellent biocompatibility.	Slow microbial growth, low yield, strict sterile conditions, and incomplete understanding of the synthesis mechanisms.	[85]

## 3. Analysis at Cellular and Molecular Scale: Uptake and Cytotoxicity of MNPs

At the cellular level, MNPs can interact with individual cancer cells through a series of physicochemical processes that include membrane binding, endocytosis, intracellular trafficking, and may trigger biological responses to eventually kill tumor cells. The cellular internalization mechanisms of nanoparticles are generally similar in different solid tumors. However, the hostile and heterogeneous TME of some tumors can substantially alter nanoparticle accessibility, distribution, and subsequent cellular uptake. To overcome these obstacles, MNPs should maintain high colloidal stability under physiological conditions and be functionalized with targeting ligands that promote receptor-mediated uptake by cancer cells [86].

### 3.1. Mechanisms of MNP Cellular Uptake

Cellular uptake represents a critical step for successful tumor-cell targeting by MNPs. The extent of MNP intracellular accumulation depends on multiple factors, including receptor-mediated endocytosis, surface chemistry, colloidal stability in biological environments, and intracellular trafficking mechanisms, which collectively determine cellular internalization, subcellular localization, and retention of nanoparticles within cancer cells.

RME is a commonly used mechanism to achieve efficient cellular uptake [87]. MNPs are recognized by specific receptors on the cell membrane through surface-bound ligands, which can eventually trigger endocytosis and induce intracellular MNP accumulation. For example, the transferrin receptor 1 (TfR1/CD71) is highly expressed in some cancer cells such as small cell lung cancer (SCLC) cells, up to 30-fold compared with other cell types [88]. SPIONs modified with the TfR1/CD71 ligand have been reported to exhibit significantly higher uptake in SCLC compared with non-modified nanoparticles in a time-dependent manner [88]. Transferrin can be conjugated with the antibody Fc fragment to prolong circulation time and improve biodistribution, particularly for applications involving blood–brain barrier transport, where TfR1 is also highly expressed [89]. However, limited accessibility of Fc fragments in the cells, random ligand orientation on the MNP surface, and poor circulation stability of MNPs may reduce targeting efficiency [90]. MNPs modified with other specific ligands such as folic acid [91], luteinizing hormone-releasing hormone (LHRH) peptides [92], or antibodies can also bind to receptors on cancer cells and enable RME. To improve ligand presentation and targeting efficiency, strategies such as stable conjugation, hydrophilic coatings, antibody fragments, or antibody immobilization can be applied [93,94].

Beyond promoting receptor-mediated uptake, surface ligands and coatings also influence nanoparticle stability and behavior in biological fluids. Surface coatings play an important role in colloidal stability and cellular interactions. Low molecular weight ligands such as citrate are often unstable under physiological conditions and may detach in the presence of divalent cations like Ca^2+^ and Mg^2+^ [95], leading to MNP aggregation. In contrast, polymer coatings such as polyacrylic acid (PAA) can provide electrostatic and steric stabilization, maintaining nanoparticles in a dispersed state and reducing nonspecific interactions [96]. To enhance water solubility, hydrophobic nanoparticles can be encapsulated within amphiphilic PEG-PCL polymeric nanocarriers using the solvent evaporation method, a highly effective, emulsion-based technique [97,98,99]. Dissolving both the nanoparticles and the PEG-PCL polymer in a volatile organic solvent, results in a mixture where the hydrophobic poly(ε-caprolactone) (PCL) blocks cluster the nanoparticles in the inner core, while the hydrophilic methoxypoly(ethylene glycol) (mPEG) chains form a water-soluble outer shell.

Particle physicochemical properties, including size, shape, and surface charge, can also influence cellular uptake efficiency. Experimental evidence suggests that nanoparticles with a radius range of approximately 25–30 nm show the best uptake efficiency, although the optimal range also depends on ligand density and surface properties of the MNPs (e.g., electrical charge) and receptor density on the target cell types. This size-dependent behavior is attributed to efficient membrane wrapping and receptor-mediated internalization with medium-size MNPs [97]. Smaller nanoparticles show reduced membrane interaction due to Brownian motion and lower ligand density-dependent affinity, whereas larger particles require more energy for their internalization and can bind more receptors, eventually leading to receptor depletion that further reduces cellular internalization. Interestingly, highly anisotropic structures, such as nanochains, can have enhanced uptake through multivalent interactions with the cell membrane [98]. Moreover, beyond regulating uptake efficiency, nanoparticle surface chemistry can directly influence cellular behavior. For instance, certain surface-modified nanoparticles like carboxyl-functionalized MNPs have been reported to have the ability to alter the cytoskeletal organization, leading to increased cellular permeability and cell death [99]. Another example is the RGD (Arg–Gly–Asp) modification. The latter can enhance the ability of MNPs to interact with integrins, thereby modulating cellular adhesion, proliferation, and signaling pathways [100].

### 3.2. Pathways of MNP Endocytosis

MNP internalization is governed by particle size, shape, surface chemistry, and the phenotype of the target cell. Across mammalian systems, MNPs enter cells through multiple endocytic pathways, each associated with distinct intracellular trafficking outcomes [101]. After the initial cellular recognition process, MNPs may be internalized, particularly in professional phagocytes, through macropinocytosis and phagocytosis, promoting rapid clearance and accumulation in the liver and spleen [102]. Non-professional phagocytes, including most non-immune and cancer cells, can perform these processes but with markedly lower efficiency [101]. Classical phagocytosis therefore plays only a minor role in standard tumor models and is generally restricted to very large particles or specialized tumor phenotypes. However, macropinocytosis becomes a dominant uptake route in KRAS-mutated cancers, where oncogenic signaling hyperactivates membrane ruffling and fluid-phase engulfment to enhance nutrient and nanoparticle uptake.

In most non-immune and cancer cell types, MNP internalization occurs primarily through clathrin-mediated endocytosis [103], followed by caveolae-dependent endocytosis as a secondary pathway [104]. Clathrin-mediated endocytosis is initiated when ligand–receptor interactions recruit clathrin triskelions and adaptor proteins, driving membrane invagination and formation of clathrin-coated vesicles that deliver MNPs to early endosomes. Caveolae-mediated endocytosis is a clathrin-independent route highly sensitive to nanoparticle dimensions. Caveolae preferentially internalize particles <60–80 nm, though they can accommodate sizes across the 20–100 nm range depending on membrane compliance [105]. These cholesterol- and sphingolipid-rich invaginations, formed by caveolin and cavin proteins, can internalize multiple small particles or a single ~100 nm MNP. Caveolae may fuse with early endosomes or mediate transcytosis across endothelial barriers, and their scission can be dynamin-dependent or dynamin-independent depending on cargo and cell type [106]. Surface chemistry and protein corona formation strongly influence caveolar uptake, often enhancing endothelial transcytosis in a size-selective manner [101]. Ligand-based functionalization—such as albumin coatings—promotes caveolar entry via gp60 binding, with up to 70% of albumin-coated nanoparticles co-localizing with caveolin-1 [106]. Certain engineered nanostructures (e.g., silica nanoparticles) can exploit caveolin-initiated macropinocytosis, where caveolin is essential for initial adhesion and membrane engulfment [106]. In addition to classical clathrin-mediated endocytosis, caveolae-mediated endocytosis, macropinocytosis, and phagocytosis, several clathrin-independent endocytosis (CIE) pathways may also contribute to the cellular internalization of MNPs, particularly for small-sized particles and ligand-functionalized systems [101].

These include the glycosylphosphatidylinositol-anchored protein-enriched compartments (CLIC/GEEC) pathway, fast endophilin-mediated endocytosis (FEME) [107], and pathways regulated by RhoA or ARF6. The CLIC/GEEC pathway is a high-capacity endocytic route regulated by CDC42 [108], GRAF1, galectins, and other associated factors, and is primarily involved in the uptake of glycosylphosphatidylinositol-anchored proteins and fluid-phase cargo. FEME is regulated by Endophilin A2 [99] and generally involves dynamin-mediated membrane scission. This pathway can be rapidly activated following ligand stimulation of specific G protein-coupled receptors or receptor tyrosine kinases and may therefore contribute to the receptor-dependent uptake of ligand-functionalized nanoparticles. RhoA-regulated CIE is generally dynamin-dependent and is influenced by Rho-family small GTPases and the composition of membrane lipid rafts. Cdc42-regulated pathways depend on actin remodeling and can generate clathrin- and caveolin-independent membrane invaginations that mediate the uptake of fluid-phase cargo and certain nanoparticles. ARF6-regulated CIE is mainly involved in the transport of specific membrane proteins and extracellular cargo to early and recycling endosomes.

Nonspecific fluid-phase uptake, traditionally classified as pinocytosis, may also contribute to the internalization of small MNPs. However, its definition and underlying molecular mechanisms overlap to some extent with those of other CIE pathways. Overall, the utilization of specific CIE pathways by MNPs is not determined by particle size alone but is collectively influenced by particle shape, surface charge, ligand density, protein corona composition, cell type, and receptor-expression profile. Accordingly, the involvement of a particular endocytic pathway should ideally be validated using a combination of complementary approaches, including pathway-selective inhibition, knockdown or knockout of key regulatory proteins, and intracellular colocalization analyses.

Once internalized, MNPs are mainly localized in endosomes and lysosomes, where they remain compartmentalized rather than freely distributed in the cytosol [109]. This intracellular route presents a major limitation for the delivery of sensitive cargos such as mRNA, which degrade in the acidic lysosomal environment (pH ~4–5). To circumvent lysosomal entrapment, modern nano-engineering incorporates biochemical and physical strategies that actively promote endosomal escape. Biochemical strategies for promoting endosomal escape involve membrane destabilization, proton buffering, and biomimetic delivery. Membrane fusogenic lipids and peptides such as DOPE and pH-responsive peptides (TAT, HA2, GALA) can induce conformational changes in acidic endosomes, enabling membrane destabilization and fusion [110]. Notably, even MNPs optimized with fusogenic liposomes, which do not rely on endosomes for internalization, still show increased lysosomal localization at later time points [111]. Amine-rich polymers, such as PEI, promote endosomal escape through proton buffering: by absorbing incoming protons in acidifying endosomes, they trigger compensatory ion influx (e.g., Cl^−^) and water accumulation, ultimately driving osmotic swelling and potential vesicle rupture, that is, the classical “proton-sponge effect” [112]. In addition, biomimetic EV–MNP hybrids may enhance cytosolic delivery through membrane interactions and modulation of intracellular trafficking [113].

Chemically driven escape mechanisms suffer from limitations such as low intracellular activation efficiency, susceptibility to enzymatic degradation, and premature cargo leakage. In contrast, the physical response of MNPs to external fields has emerged as a powerful strategy for facilitating endosomal and lysosomal escape. These methods offer significant advantages, particularly high spatiotemporal controllability and reduced reliance on intracellular chemical reactions. Magneto-mechanical disruption is a representative strategy among these approaches. Under a low-frequency alternating magnetic field, MNPs can oscillate, rotate, or generate torque within the endosomal or lysosomal lumen [114]. The resulting mechanical stress induces local shear forces and membrane deformation, creating transient permeability or physical rupture in the endosomal/lysosomal membrane, thereby facilitating the release of encapsulated therapeutic cargo into the cytoplasm [115]. Localized nanoscale heating represents another physical mechanism for achieving intracellular cargo release. Upon activation by magnetic fields or photothermal stimuli, heat generated near the nanoparticle surface destabilizes the lipid bilayer of the endosomal or lysosomal membrane and increases membrane permeability. This highly localized thermal effect promotes rapid membrane rupture and cargo release into the cytoplasm while keeping the macroscopic temperature rise minimal. Osmotic swelling of endosomes can also trigger physical membrane rupture. The accumulation of ions at high concentrations within the endosomal lumen increases osmotic pressure and drives water influx, leading to endosomal swelling [116].

### 3.3. Mechanisms for MNP Cytotoxicity and Antitumor Effects

Magnetic hyperthermia and stimulus-responsive drug delivery represent the main therapeutic modalities enabled by MNPs. Under an alternating magnetic field, MNPs convert magnetic energy into heat, producing temperature-dependent cytotoxic effects. Severe hyperthermia (>45 °C) induces irreversible protein denaturation, membrane disruption, and cell death, resulting in thermal ablation of tumor tissues. Mild hyperthermia (~42 °C), instead, perturbs membrane integrity and intracellular homeostasis, thereby enhancing the cytotoxicity of chemotherapeutic agents and radiotherapy [117].

Beyond these thermal effects, MNPs can also induce cytotoxicity through non-thermal mechanisms. Under external magnetic fields, anisotropic magnetic nanostructures—such as nanowires, nanodiscs, and nanospikes—can generate mechanical forces, such as vibration and torque, capable of disrupting cellular membranes [118], including lysosomal compartments [114], thereby inducing or facilitating cell death. In addition to these direct mechanical effects, MNP-induced cytotoxicity can also involve the magnetomechanochemical effect, in which magnetic nanostructures undergo electronic changes when exposed to combined static magnetic fields and AMF [119]. These changes can promote the transfer of unpaired electrons from nanoparticles to surrounding molecules—including endogenous biomolecules and exogenous chemotherapeutic agents—leading to the formation of reactive species capable of modifying molecular bonds and triggering cascades of chemical reactions. A particularly relevant pathway involves ROS generation through the Fenton reaction [120]. In acidic compartments such as lysosomes, ferrous ions (Fe^2+^) released from MNPs react with endogenous hydrogen peroxide (H_2_O_2_), producing highly reactive hydroxyl radicals (•OH) [121]. These radicals induce oxidative damage to lipids, proteins, and DNA. Excessive iron and ROS accumulation can trigger ferroptosis [122], a non-apoptotic form of programmed cell death driven by lipid peroxidation, which may help overcome drug resistance. Particle size, chemical composition, crystal phase, and the local microenvironment critically influence iron release, Fenton-like activity, and ROS production [123]. At the organelle level, ROS can impair mitochondrial membranes and disrupt Ca^2+^ homeostasis, reducing membrane potential and amplifying oxidative stress, ultimately leading to mitochondrial dysfunction and cell death [124,125].

**Figure 2:**
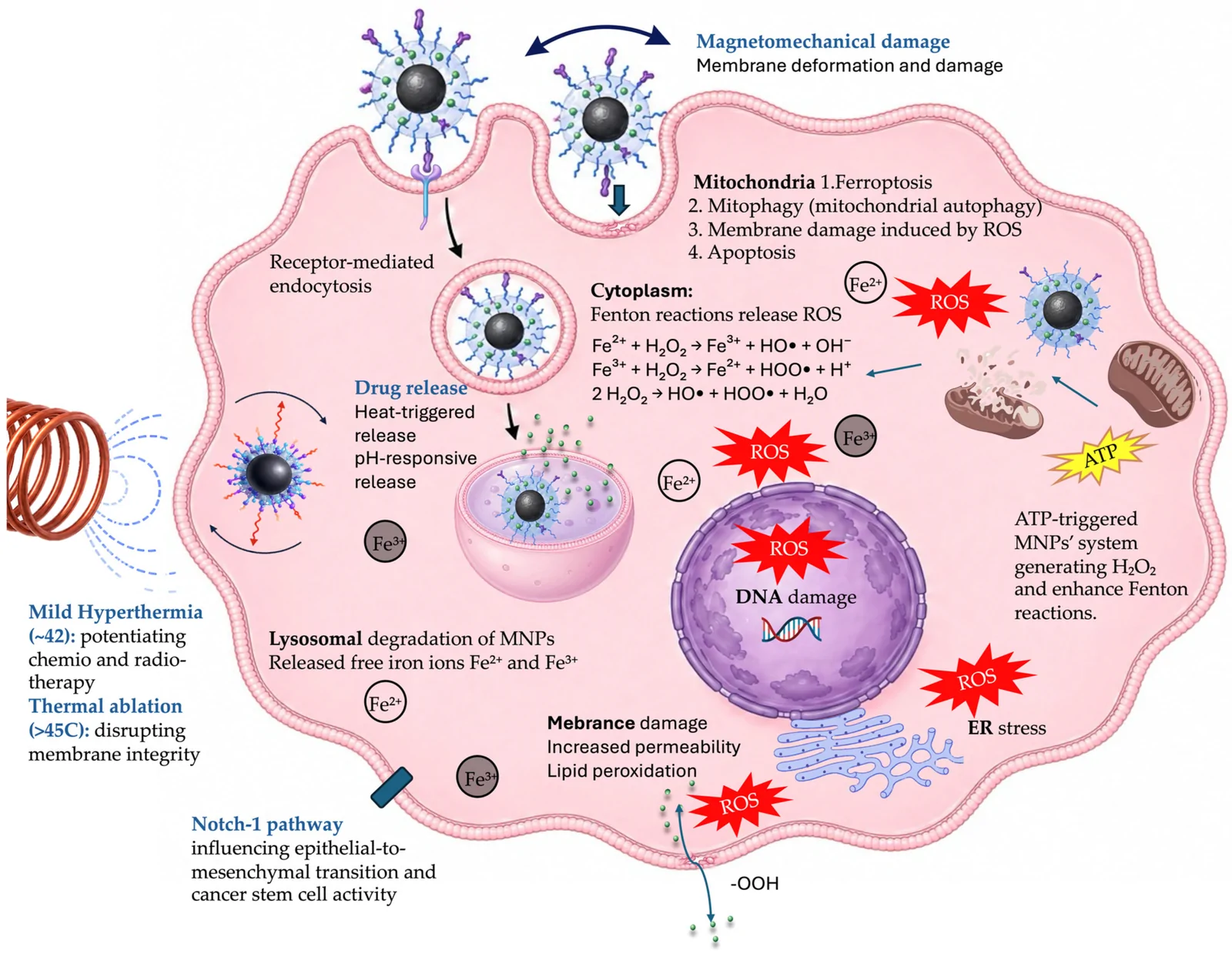
Cellular uptake and intracellular therapeutic mechanisms of MNPs.

MNPs can also function as smart nanocarriers capable of releasing chemotherapeutic agents in response to specific stimuli, thus enabling spatially controlled drug delivery and increasing local drug availability. MNPs can engage in intracellular biochemical interactions and modulate signaling pathways [126] (Figure 2). Cargo release from MNP carriers can be triggered by diverse intracellular mechanisms, depending on the carrier architecture and its subcellular localization. Chemical stimuli such as acidic pH, lysosomal enzymes, reactive oxygen species (ROS), or glutathione (GSH)-mediated bond cleavage, as well as physical triggers such as magnetic heating, can initiate drug liberation. For example, ROS-responsive nano-prodrugs undergo intracellular dissolution after endocytosis, followed by H_2_O_2_-induced chemical conversion and lactonization, ultimately releasing the active drug that mediates cytotoxicity [111]. However, tumor cell heterogeneity complicates intracellular delivery. Distinct cancer cell subpopulations may differ in receptor expression, receptor density, membrane biophysical properties, and preferred endocytic pathways. Consequently, even within a single tumor, these differences lead to heterogeneous MNP uptake and uneven intracellular accumulation, resulting in variable therapeutic responses. Ligand–receptor binding not only facilitates cellular uptake but can also activate or inhibit receptor-dependent signaling. For example, targeting the Notch-1 receptor suppresses its signaling, influencing epithelial-to-mesenchymal transition and cancer stem cell activity [127], whereas engaging the stimulator of interferon genes (STING) pathway enhances type I interferon signaling [128]. Although signaling-modulation strategies are still at an early stage and currently involve a limited number of targets, they hold considerable potential as new molecular regulators are identified and ligand-coated MNPs are further optimized. Other targeting mechanisms exploit specific properties of the tumor environment to selectively release drugs. Nutrient and oxygen supply inside many tumors is insufficient, and the TME is characterized by relatively acidic conditions due to enhanced glycolysis and lactate accumulation. In solid tumors, extracellular pH can be around 6.2–6.8 compared to normal 7.2–7.4 [129]. Such an acidic environment provides a unique opportunity for the design of pH-responsive MNP systems, which can achieve controlled and site-specific drug release based on local pH variations, thereby improving targeting efficiency [130]. Drugs such as cisplatin can be conjugated to nanocarriers through pH-sensitive linkers and released in response to acidic conditions within the TME [131]. The efficiency of the pH-targeting method is strongly dependent on tumor volume and vessel distribution [132]. Drug release can also be triggered by enzymatic cleavage or redox-induced bond cleavage in the TME. For instance, in tumor stroma, matrix metalloproteinase (MMP) activity is high. Targeting methods also include matrix metalloproteinase targeting, which can selectively release drugs inside the tumor [133].

Besides drug delivery, TME-responsive biochemical reactions can also act as a trigger of programmed cell death in the absence of chemotherapeutic drugs. E.g., For example cancer cells exhibit increased glucose uptake coupled with the Warburg effect, causing abnormal diffusion of glucose in the TME [134]. This increased consumption of glucose can serve as a metabolic target. For example, MNPs equipped with the GSH-responsive biomimetic hybrid nanoenzyme system M@GOx/Fe-HMON (where hollow mesoporous organosilica nanoparticles encapsulate glucose oxidase (GOx) and Fe^2+^/Fe^3+^ couples, while the outer region is M-cloaked with cancer cell membranes, to allow for tumor targeting and immune evasion) can be activated by high intratumor GSH levels to release GOx, which in turn induces glucose depletion, starving cancer cells and turning off the Warburg effect. Moreover, glucose oxidation also produces large amounts of H_2_O_2_, which can be used by the Fe-containing core to initiate the Fenton reaction and produce highly damaging hydroxyl radicals [135], while acidifying the environment [136]. This method also addresses the issue of limited efficacy caused by insufficient H_2_O_2_ in the TME for the Fenton reaction [137]; combined with the high drug release efficiency of MNPs in acidic environments, it may represent a combined targeted therapeutic approach for tumors such as some gliomas [129].

MNPs can also be used to modulate autophagy in cancer therapy. Nanocarriers may deliver autophagy regulators together with chemotherapeutic agents, promoting intracellular accumulation and synergistic cytotoxicity [138]. Oxidative stress and disruption of mitochondrial electron and proton transport can further reduce ATP production, causing energy depletion and contributing to cell death—providing an additional mechanism beyond conventional drug-based approaches. Despite these cytotoxic mechanisms, therapeutic efficacy can be limited by the intrinsic heterogeneity of cancer cells, including multidrug resistance (MDR) phenotypes [139]. Cancer stem cells (CSCs) can evade therapy through dormancy and later drive tumor recurrence [140]. CD44-targeted RHID (ICG-DOX@RA-HA-DOX) nanocomplexes, with a hyaluronic acid–retinoic acid–doxorubicin (RA-HA-DOX) shell, have been investigated as a strategy to promote CSC differentiation and reduce recurrence risk [141].

Overall, MNPs can exert anticancer effects through multiple interconnected pathways. However, as with conventional chemotherapeutics, insufficient tumor selectivity and off-target accumulation may also affect normal tissues, leading to adverse effects. To better evaluate cytotoxicity, 3D tumor spheroids and organ-on-chip platforms have been employed. Since MNP efficacy strongly depends on tissue penetration and cellular uptake, these advanced models provide a more realistic assessment of therapeutic performance than traditional 2D cultures [142,143].

Following receptor-mediated endocytosis, MNPs can induce therapeutic effects through multiple pathways, including magnetothermal activation (hyperthermia with different target temperatures), ROS generation via Fenton reactions, lysosomal degradation and iron ion release, mitochondrial dysfunction, ferroptosis, ER stress, DNA damage, and modulation of cellular signaling pathways such as Notch signaling. Heat-mediated, ligand binding-induced, or lysosome-activated drug release can also happen at the same time.

## 4. Delivery at the Whole-Body Scale: Targeted Delivery of MNPs to Tumors

On the whole-body scale, the magnetic properties of MNPs enable remote control of their motion and heating through electromagnetic systems such as Helmholtz coils, Maxwell coils, and high-frequency solenoids. External magnetic fields can also modulate the magnetic relaxation mechanisms of MNPs, thereby regulating temperature increases and allowing selective heating of specific anatomical sites [144]. This electromagnetic actuation and targeting can synergize with molecular targeting strategies—such as antibody-mediated recognition of specific tumor antigens—and with intracellular mechanisms like endosomal escape, jointly enhancing therapeutic efficacy [145].

There are biological factors that facilitate MNP tumor accumulation, but also multiple barriers that limit their effective delivery and increase the risk of off-target toxicity, making it necessary to develop dedicated strategies to overcome them. The TME is characterized by irregular vasculature and poor lymphatic drainage, conditions that promote nanoparticle extravasation while simultaneously hindering their clearance. This phenomenon, known as the enhanced permeability and retention (EPR) effect, cooperating with other targeting mechanisms, enables MNPs to accumulate at tumor sites, where they can exert cytotoxic effects. In contrast, healthy tissues typically exhibit lower MNP accumulation and more efficient heat dissipation through physiological perfusion. As a result, they experience reduced temperature elevation during exposure to magnetic or radiofrequency fields and are less susceptible to additional cytotoxic mechanisms of MNPs. This limits damage to healthy tissues, although off-target effects remain an important safety concern [146].

The downside of the EPR effect, however, is that it is highly heterogeneous, potentially leading to uneven distribution. This is related to the fact that TME has physical features characterized by differential stromal stiffness depending on tumor type and stage. For example, the dense extracellular matrix (ECM) in fibrous tumors [147] can restrict nanoparticle diffusion. Even within the same patient, different levels of elevated interstitial fluid pressure and leaky blood vessels may inhibit the ability of MNPs to penetrate into the tumor core, resulting in heterogeneous intratumoral distribution and less efficient therapeutic outcomes [148]. Hypoxic regions located far from functional vessels are particularly difficult for MNPs to reach. Moreover, acidic pH may alter particle stability, while infiltrating immune cells, particularly tumor-associated macrophages, may either transport or sequester MNPs. Thus, abnormal vasculature, ECM stiffness, hypoxia, acidity, and immune cell infiltration collectively produce heterogeneous MNP delivery. Also, the EPR effect exhibits significant clinical and pathological differences between humans (clinical patients) and animal models (primarily rodents), leading to unsatisfactory clinical outcomes for many nanomedicines that perform exceptionally well in animal experiments [149]. The main differences lie in the tumor-to-body ratio and vascular structure and heterogeneity. Subcutaneous xenografts in mice grow extremely rapidly, increasing by approximately 1 cm within 2–4 weeks. In mice, tumor volume and mass can reach up to 10% of body weight, equivalent to a watermelon-sized tumor in a 70 kg human [150]. This high proportion makes mouse tumors a large drug filter and accumulation pool, artificially enhancing drug efficacy and reducing toxicity. Due to differences in blood perfusion kinetics, after body weight normalization, mice expose tumors to circulating nanomedicines at a level approximately 100 times higher than human patients [151]. In mouse models, the accumulation concentration of nanomedicines in tumors typically decreases with the increase in tumor volume. However, in human patients, enhanced penetration was still observed in about half of the patients with large tumors, while medium-sized tumors showed increased penetration in the vast majority of cases [152]. Furthermore, animal models are highly homogeneous. Most mouse xenografts used in experiments are grown from a single cell line in a short period, resulting in highly homogeneous cellular components, which might be favorable for traditional EPR effects and nanodrug accumulation. Clinical human tumors, in contrast, are extremely complex. Human tumors grow slowly under prolonged immune pressure and exhibit high genetic and structural heterogeneity. Their vascularization and endothelial gaps are uneven; some areas may completely lack permeability fenestrations, while others have complete pericyte coverage [153], making it difficult for drugs to penetrate blood vessels. In addition, human tumors (such as pancreatic cancer) typically possess extremely dense and compact ECM [154], which constitutes a strong physical barrier. With the dense proliferation of cancer cells, the interstitial hydraulic pressure (IFP) within the tumor significantly increases. This creates an inward–outward pressure gradient, hindering the penetration of nanoparticles and forcing drug delivery to rely on inefficient diffusion [155] rather than the more efficient transport found in mice. In human clinical tumors, abnormal vascular structure and uneven blood flow are commonly accompanied by thrombosis and occlusions, which greatly hinder the delivery of nanomedicines in the human body [156]. However, these are rarely observed in mouse models.

A complex and constantly changing TME and the hard-to-pass immune system and in vivo barriers create the demand for a dynamic tunable material that can adjust to different environments. Studies have shown that current surface functionalization methods can provide nanocarriers with at least partial control over their pharmacokinetics and biodistribution [157]. This may lead to higher tumor uptake and greater tumor eradication with no apparent systemic or off-target toxicity [158]. Moreover, cooperative approaches combining passive and active targeting can be used. Active targeting at the cellular level can be achieved by surface-conjugating specific ligands, such as LHRH receptors [159], EpCAM [160], or the more tumor-selective EpCAM homolog Trop2 [161], thereby increasing the accumulation of particles at the correct lesion site.

The physiological environment significantly challenges ligand stability. High ionic strength, abundant endogenous biomolecules, and enzymatic activity can compromise surface functionalization. These factors include ligand desorption and exchange, enzymatic degradation, and premature cargo release. Ligand desorption and exchange occurs because weakly adsorbed ligands or electrostatic interactions often dissociate upon contact with plasma proteins. Thiol-based linkages (e.g., Au–S bonds) are particularly vulnerable to displacement by endogenous GSH or albumin. Enzymatic degradation occurs when peptides, proteins, and aptamers are continuously exposed to proteases and nucleases, which can eliminate the targeting capability before MNPs reach the diseased tissue. Premature cargo release occurs when stimuli-responsive linkers degrade under physiological pH (~7.4), causing off-target drug leakage and systemic toxicity. To mitigate these issues, modern designs increasingly rely on covalent conjugation strategies, including click chemistry and carbodiimide coupling (EDC/NHS), which provide robust ligand retention during circulation.

Cell membrane-coated MNPs represent a transformative approach to nanoparticle stealth and targeting. By wrapping synthetic cores with natural membranes derived from red blood cells, leukocytes, platelets, or cancer cells, these hybrid nanosystems acquire the complex antigenic landscape and functional properties of the source cells [162]. RBC-coated MNPs exploit CD47, a self-recognition marker that binds to SIRPα receptors on macrophagess to deliver a “do not eat me” signal, suppressing phagocytosis and substantially prolonging circulation half-life [162]. 

Leukocyte and platelet membrane coatings confer natural inflammation-homing capabilities, enabling MNPs to traverse damaged endothelium and accumulate in inflamed or ischemic tissues. Tumor-derived membrane coating enables homotypic targeting, driven by adhesion molecules such as N-cadherins and EpCAM, allowing MNPs to selectively bind cells of the same tumor type. Their multi-antigenic profile also facilitates immune evasion [163]. Importantly, membrane coatings shield the nanoparticle core from direct plasma protein adsorption, replacing the synthetic protein corona with a pre-formed native layer. This minimizes opsonization, reduces MNP clearance, and enhances translational potential.

Moreover, in contrast to professional phagocytes, tumor cells lack a functional reticuloendothelial system (RES) and therefore do not possess the highly efficient, nonspecific phagocytic machinery found in Kupffer cells or splenic macrophages. As a result, nanoparticles without prolonged circulation features (e.g., PEGylation) or active targeting ligands are rapidly sequestered and cleared by RES macrophages before reaching tumor tissue. This systemic filtration means that only a small fraction of the injected dose is available for tumor uptake [164].

As mentioned before, the formation of a protein corona affects MNP cellular uptake. Protein corona also influences systemic biodistribution and can promote nonspecific recognition and clearance by immune cells. However, MNPs can also, based on their own properties and independently of a protein corona, actively modulate the tumor immune microenvironment. For instance, magnetic Janus particles coated with FePt nanolayers have been shown to interact with tumor-associated macrophages (TAMs) and induce their polarization from the tumor-promoting M2 phenotype toward the tumor-suppressing M1 phenotype [165]. Polarization-reversed M1 macrophages release pro-inflammatory cytokines (such as ROS and TNF-α), directly participating in the killing of cancer cells and activating the immune response. The extent and composition of tumor-infiltrating immune cells can substantially influence MNP transport and intratumoral distribution, thereby affecting their accessibility to cancer cells. Highly phagocytic myeloid cells, particularly TAMs, can sequester nanoparticles and reduce direct cancer-cell exposure [166], whereas migratory macrophages and neutrophils can also act as cellular carriers that enhance vascular crossing and deeper intratumoral transport [167]. Furthermore, recent studies have demonstrated that MNP-based platforms can also be used to trigger immune-mediated tumor suppression. For instance, multifunctional systems such as αPD-L1/CpG-loaded MNPs have been shown to promote the activation of CD8^+^ T cells [168], which can directly kill tumor cells. Under the influence of a rotating magnetic field (RMF), MNPs can also function as microrobots, inducing a state change in antigen-presenting cells (APCs), for example in dendritic cells (DCs). Biomimetic MNPs can cause an up-regulation of the maturation markers CD80, CD83, and CD86 in immature monocyte-derived DCs, stimulating DC maturation [169]. These immune-regulation capabilities may serve as an alternative strategy to address drug resistance and potentiate immunotherapy.

In terms of tumor treatment, MNPs have shown considerable advantages over traditional chemotherapy, including lower systemic toxicity and the ability to move efficiently through micro-vessels [170,171]. Most microvascular endothelial barriers permit the passage of MNPs smaller than ~150 nm in diameter; however, more restrictive interfaces—particularly the blood–brain barrier (BBB)—remain difficult to cross. The BBB has long represented a major obstacle for drug delivery to the central nervous system. To improve MNP transport across this barrier, several strategies have been explored, including surface functionalization with targeting antigens such as TfR1/CD71 [89], ultrasound-assisted delivery, and electromagnetic actuation [172]. Targeting ligands like TfR1/CD71 facilitate receptor-mediated transcytosis, enabling MNPs to bind to and traverse brain endothelial cells. Beyond biochemical targeting, MNPs offer the unique advantage of being actuated by external magnetic fields, which can facilitate the targeted accumulation of MNPs within tumors. However, magnetic targeting does not fully resolve the challenge of deep tissue penetration. Animal studies indicate that external magnetic fields can increase intratumoral levels of iron oxide nanoparticles (IONPs), although overall targeting efficiency after systemic administration remains limited, with a substantial fraction of particles still being cleared by the liver. Developing magnetically actuated nanorobotic systems with active navigation capabilities may represent a promising solution to overcome these limitations.

Compared with traditional chemotherapy, drugs delivered through MNP-based systems can achieve stronger therapeutic effects with lower systemic toxicity. MNP-mediated drug delivery has proven feasible and effective in multiple animal tumor models [173], where nanoparticles often outperform conventional chemotherapeutics while causing fewer adverse effects [174]. Similar results have been observed in vitro, with MNP-delivered drugs showing enhanced anticancer activity compared to their free form [175]. Drug efficacy is frequently limited by nonspecific biodistribution and rapid metabolic and renal clearance [176]. MNPs can improve these parameters by selectively transporting therapeutic agents to tumors, slowing their release rate, and prolonging their bioavailability while reducing premature degradation and off-target accumulation [177]. As multifunctional platforms are increasingly needed to overcome the limitations of standard cancer therapies, MNPs are emerging as a promising therapeutic tool. For example, amine-functionalized nanocrystalline cellulose grafted with folic acid and Fe_3_O_4_ nanoparticles (AF-NCC/Fe_3_O_4_) has been used to enable targeted delivery of doxorubicin and reduce cardiotoxicity compared with conventional administration [178].

Overall, the key advantage of MNPs in whole-body delivery lies in their ability to target the tumor microenvironment, modulate immune responses, and be remotely actuated to optimize intratumoral distribution and selectively induce therapeutic effects such as hyperthermia and controlled drug release. These features support precise, contactless treatment of deep tissues, making MNPs a promising and attractive option for tumors that are difficult to reach surgically.

## 5. Clinical Applications and Therapeutic Evaluation of MNPs: Status of the Art

The potential clinical utility of MNPs depends on several tumor-specific characteristics, including vascularization, anatomical location and depth, and extracellular matrix composition. Pancreatic cancer, for example, is characterized by a dense, CAF-driven stromal microenvironment that contributes to immunosuppression and resistance to conventional therapies. In this context, MNP-based approaches are being investigated as potential complementary therapeutic strategies [179]. Other tumor types may benefit from different properties of MNP platforms. In breast cancer, the relatively superficial location of many lesions may facilitate the application of external magnetic fields. Malignancies such as locally recurrent prostate cancer, hepatocellular carcinoma, melanoma, and ovarian cancer often present with multifocal or disseminated disease that can complicate surgical management. In these settings, the ability of MNPs to be magnetically guided and to provide localized hyperthermia or controlled drug release may offer a minimally invasive approach for targeting lesions that are difficult to treat surgically [180]. MNP-based therapies may also be particularly valuable in tumors for which standard treatment options are limited by anatomical constraints or by the risk of treatment-related toxicity, while effective magnetic guidance is feasible. Brain gliomas represent a notable example, as their location, the sensitivity of surrounding neural tissue to cytotoxic drugs, and the restrictive nature of the blood–brain barrier all limit the effectiveness of conventional therapeutic approaches. At the same time, the altered metabolic microenvironment characteristic of these tumors may provide endogenous stimuli for the activation of appropriately engineered MNP platforms [181]. Finally, in cancers such as lung cancer and osteosarcoma, where extensive surgical intervention may compromise organ or limb function, MNPs are being investigated as adjuncts to established therapies. In these applications, magnetic hyperthermia can provide localized heating of tumor tissue, while imaging-based monitoring and thermometry techniques may support treatment guidance and assessment.

In the following sections, we discuss routes of MNP administration, methods for nanoparticle activation, approaches for quantifying therapeutic efficacy, and the role of predictive modeling in supporting nanoparticle design and treatment planning.

### 5.1. Administration of MNPs

**Figure 3:**
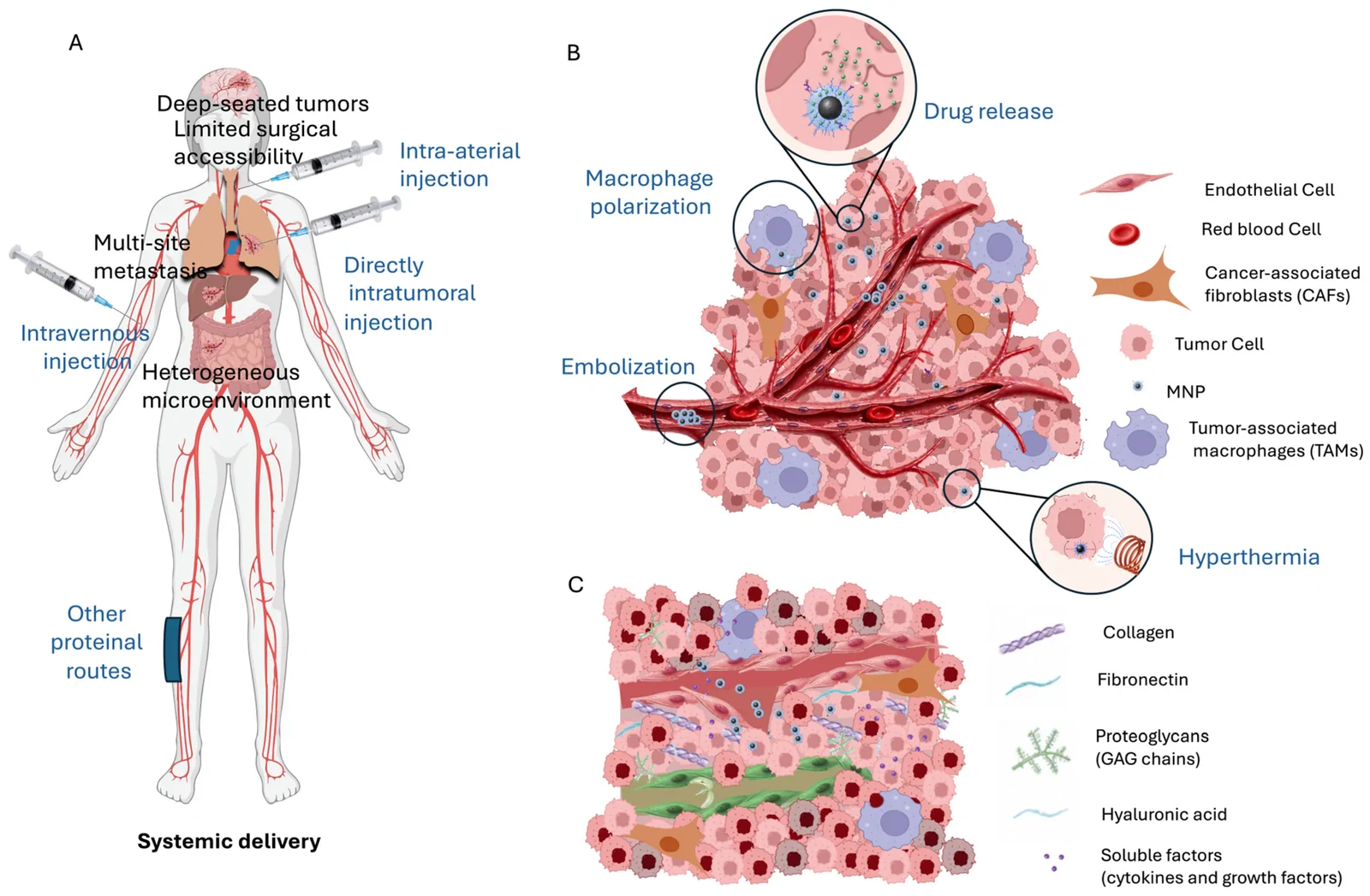
Administration of MNPs. (**A**) Different administration routes for MNP delivery in inoperable cancer. (**B**) Interactions between MNPs and the tumor microenvironment. (**C**) Structural components and EPR in tumor stroma.

MNPs can be administered to patients as colloidal suspensions or other formulations, most commonly by intravenous injection. Following administration, external magnetic field gradients may be used to guide drug-loaded MNPs toward the target tissue. Several MNP-based formulations have been clinically approved as MRI contrast agents, enabling the tracking of injected nanoparticles in vivo [182]. Alternatively, MNPs can be delivered directly to the tumor through intratumoral injection [183]. The latter enables high local MNP accumulation and precise control over nanoparticle dosage at the target site, bypassing the low tumor accumulation that often limits systemic administration and making it one of the most efficient administration routes in both preclinical and clinical studies. Consequently, it may be particularly attractive for patients who are not candidates for radical surgical resection because of tumor location, proximity to critical vascular or neural structures, extensive disease burden, or inability to tolerate surgery. Although external magnetic guidance is not required for tumor targeting in this setting, magnetic fields may still contribute to nanoparticle retention at the tumor site. However, its applicability is largely restricted to tumors that can be directly accessed by injection. Furthermore, because MNPs are delivered into a confined region of the tumor, they may aggregate and exhibit limited intratumoral dispersion, resulting in heterogeneous nanoparticle distribution and, consequently, uneven therapeutic effects throughout the lesion [184]. Alternative administration strategies, including implantation [185], transdermal delivery via skin patches [186], and inhalation [187], have also been explored for MNP delivery (Figure 3A).

#### 5.1.1. MNPs in Embolization Therapy

The ability of MNPs to accumulate at specific sites under magnetic guidance can be exploited for embolization therapy. In this approach, interfacial roughness-reinforced MNPs are delivered through arterial or venous catheters to selectively occlude tumor-feeding vessels, thereby interrupting perfusion and inducing ischemic tumor necrosis [188]. MNP-based magnetic embolic systems, including magnetic microspheres [189] and magnetically actuated microrobots [190], are particularly attractive for the treatment of deep-seated primary and metastatic solid tumors. The extent of vascular occlusion can be tuned through particle size selection according to the diameter of the target vessels. Beyond mechanical vessel obstruction, these systems can simultaneously serve as drug carriers, promoting local drug retention within the embolized tissue, enhancing therapeutic efficacy while reducing systemic exposure and off-target toxicity. In addition, embolized tumors can subsequently be subjected to magnetic hyperthermia, while immunomodulatory effects associated with MNP accumulation may further contribute to therapeutic outcomes (Figure 3B).

An alternative strategy involves injectable magnetic nanocomposite hydrogels that form embolic structures in situ. These materials combine high injectability, hemostatic capability, mechanical robustness, wet adhesion, and magnetothermal responsiveness, making them attractive multifunctional platforms for locoregional cancer treatment. Preclinical studies have demonstrated their successful use—following minimally invasive administration by percutaneous intervention under ultrasound guidance—both in sealing surgical margins after tumor resection and in hyperthermic ablation to prevent tumor recurrence after surgery or as a primary treatment for unresectable tumors [191]. TME-responsive systems have further expanded this therapeutic platform. For example, poly[(L-glutamic acid-ran-L-tyrosine)-b-L-threonine-b-L-cysteine] (PGTTC)-based nanocomposites undergo self-assembly into a gel under acidic tumor conditions, inducing microvascular embolization and sustained perfusion blockage that results in substantial tumor necrosis and shrinkage in animal models [192]. More recently, Cu-Fe_3_O_4_ nanoclusters and artemisinin co-loaded into injectable alginate hydrogels demonstrated prolonged local release of nanocatalysts, continuous oxidative stress amplification, and significant antitumor efficacy against osteosarcoma in vivo, highlighting the potential of embolic hydrogels for the treatment of deep tumors that are not suitable for complete surgical resection [193].

Beyond vessel occlusion, embolization-induced ischemia and hypoxia can promote extensive ECM remodeling [194]. However, the impact of these microenvironmental changes on intratumoral particle distribution and therapeutic efficacy remains largely unexplored (Figure 3C).

From a materials perspective, magnetic embolic agents are commonly fabricated using hydrophilic biocompatible polymer matrices such as PAA [195], poly(lactic-co-glycolic acid) (PLGA) [196], and fatty amide-based polyurethane urea (PHEUU) [197]. These materials minimize systemic leakage while allowing controlled biodegradation when temporary occlusion is desired. Their spherical morphology and favorable mechanical properties such as compressibility and elasticity facilitate catheter-based delivery, allowing them to withstand deformation during vascular transport, while enabling effective vascular obstruction. Depending on the degradable or non-degradable polymer composition of the coating material, either transient or permanent embolization can be achieved. Furthermore, the intrinsic MRI contrast properties of MNPs allow real-time visualization of embolic distribution, embolization density, and procedural endpoints.

Despite these advantages, several technical challenges continue to limit clinical implementation. Reflux and off-target embolization may cause ischemic damage to healthy tissues [198], while vasospasm during catheter-based delivery, complex vascular architectures, and washout under high-shear blood flow can reduce embolization efficiency [199]. To address these limitations, balloon-assisted delivery techniques have been employed to transiently reduce blood flow and minimize reflux [200]. Moreover, more adaptive magnetically actuated microrobotic swarms can be designed and coupled with real-time MRI guidance, particularly using T2*-weighted gradient-echo sequences, offering real-time monitoring of particle distribution and facilitating early detection of reflux events during embolization and timely intervention. Additional strategies aiming to enhance local retention include the implantation of paramagnetic scaffolds or stents that create magnetic capture zones within blood vessels, where the directional trapping of MNPs can be controlled. In these systems, capture efficiency increases with magnetic field strength and particle size and decreases with flow velocity [201].

Washout by high-shear arterial blood flow remains a major limitation to MNP efficacy [199], although novel magnetic targeting approaches are being developed to enhance particle retention and guidance at greater tissue depths than currently achievable [202]. Fabricated from magnetic hydrogel inks through 3D-printing technologies, these systems can dynamically change shape under external magnetic fields, enabling active navigation and improved filling of complex vascular geometries in vivo [190]. This allows for more stable and complete embolization compared to conventional passive approaches. Nevertheless, excessive distal embolization may still result in microvascular rupture and leakage when arterioles become obstructed during capillary-targeted procedures [203]. Consequently, recent strategies increasingly focus on achieving proximal vascular occlusion of arterioles while preserving the possibility of drug delivery to distal tumor regions through capillaries.

Early studies demonstrated the feasibility of integrating 20–50 nm MNPs into embolic matrices carrying additional functions, including externally triggered drug release [204]. The prolonged retention of therapeutic agents within embolized tissues enables dose reduction and enhances local treatment efficacy [205]. Combined with magnetic targeting and hyperthermia, embolization-based approaches can maximize tumor damage while sparing surrounding healthy tissues [203]. Encouragingly, the translational potential of these systems has already been demonstrated in preclinical studies. For example, RegFe@MS magnetic microspheres have been successfully applied in transarterial chemoembolization [206], while magnetically guided embolic particles have achieved lobe-selective therapeutic effects in liver tumor models [207].

#### 5.1.2. Multimodal Imaging for Treatment Guidance and Monitoring

Multimodal imaging plays a central role in MNP-based therapies by enabling accurate tumor localization before activation, verification of nanoparticle delivery and intratumoral distribution, guidance of spatially precise activation, and monitoring of treatment response and residual disease. Before treatment, imaging can confirm whether MNPs reach the tumor and accumulate at sufficient concentrations. For example, CT-based treatment planning has been used to determine the optimal number and location of MNP reservoirs in the prostate based on patient anatomy and predicted specific uptake. Nanoparticles are then injected under transrectal ultrasound and fluoroscopic guidance, and their distribution is monitored through repeated CT scans over a six-week period, allowing multiple hyperthermia sessions without reinjection [180]. 

While CT is particularly valuable for treatment planning and monitoring nanoparticle distribution, MRI provides superior soft-tissue contrast and high-resolution anatomical information while also enabling visualization of MNP accumulation, making it particularly suitable for deep or anatomically complex tumors such as glioblastoma. Accordingly, recent preclinical studies have explored MRI-guided magnetothermal therapy in glioblastoma and other solid tumor models [208].

However, conventional MRI has limitations for quantitative treatment planning. The high iron concentrations required for magnetothermal therapy cause strong T2/T2* shortening and magnetization effects, resulting in signal loss and artifacts, which complicate accurate mapping of nanoparticle distribution or concentration. Magnetic particle imaging (MPI) addresses these challenges by directly detecting the nonlinear magnetic response of superparamagnetic nanoparticles rather than signals from surrounding tissues [209]. Therefore, MPI provides high-contrast, quantitative maps of MNP distribution without ionizing radiation. MPI-guided magnetothermal therapy can first image and quantify nanoparticle localization, then use magnetic field gradients to confine heating to selected tumor regions while minimizing off-target heating in surrounding tissues or organs that have accumulated nanoparticles [210]. Recent research has also explored MPI-guided magnetohydrodynamic thermotherapy to predict magnetothermal dose and achieve spatially localized treatment [211]. MPI is therefore particularly attractive for deep inoperable tumors that require precise control of the spatial extent of thermal activation, although clinical translation remains limited by the absence of human-scale MPI systems and suitable tracers.

During nanoparticle activation, imaging helps define the target treatment volume and reduce off-target activation. Anatomical MRI and temperature feedback from magnetic resonance thermometry [212] ensure that MNP-mediated therapies are accurately targeted to deep or surgically inaccessible tumor regions. After activation, imaging can assess treatment response, residual lesions, and changes in the tumor microenvironment. Conventional MRI can monitor tumor volume, necrosis [213], edema, and tissue architecture [214], while functional or molecular imaging can detect early treatment-related changes that precede macroscopic shrinkage [215]. Thus, multimodal imaging can not only guide treatment targeting but also support longitudinal evaluation of MNP-mediated therapeutic efficacy.

### 5.2. Activation of MNPs

A unique advantage of MNPs is their ability to be remotely activated or actuated and to exert effects selectively within targeted regions [216]. Depending on the application, MNPs can be activated through different mechanisms. Among them, the most widely known and studied is magnetothermal activation by external AC magnetic fields [217]. Magnetic hyperthermia, including magnetic fluid hyperthermia (MFH), is one of the main therapeutic applications of magnetothermal activation. In this process, activated MNPs convert magnetic energy into heat, increasing the temperature of target tissues and cells [218]. Another important approach is magneto-mechanical activation, in which MNPs convert external magnetic fields into mechanical forces or torques acting on biological structures. Unlike magnetic hyperthermia, this strategy relies on nanoparticle motion rather than heat generation to remotely regulate cellular functions and induce therapeutic effects [219]. Under low-frequency or RMFs, MNPs produce translational forces and rotational torques. Its efficiency depends on factors such as particle shape anisotropy, clustering, magnetic field gradient [220], and the properties of the surrounding medium [221]. In particular, the magnetic field gradient directly determines the magnitude of the force exerted on MNPs, thereby influencing both mechanical stimulation and magnetic targeting. However, increased viscosity can hinder nanoparticle mobility and rotation, limiting force transmission and reducing therapeutic efficacy, especially in highly constrained intracellular environments. Because it operates at low frequencies, magneto-mechanical activation minimizes eddy-current heating and may represent a safer alternative to thermal magnetic therapies.

Chemical or catalytic activation involves the initiation or enhancement of MNP-mediated catalytic reactions in response to local biological and chemical conditions. Unlike thermal or mechanical activation, it generally does not require continuous external stimulation, relying instead on TME conditions [222]. These stimuli can trigger nanoplatform degradation, metal ion release, exposure of active sites, or catalytic valence transitions, converting relatively inert MNPs into active nanozymes. Therefore, TME-responsive activation represents the main endogenous activation mode of MNP-mediated catalytic therapy.

**Figure 4:**
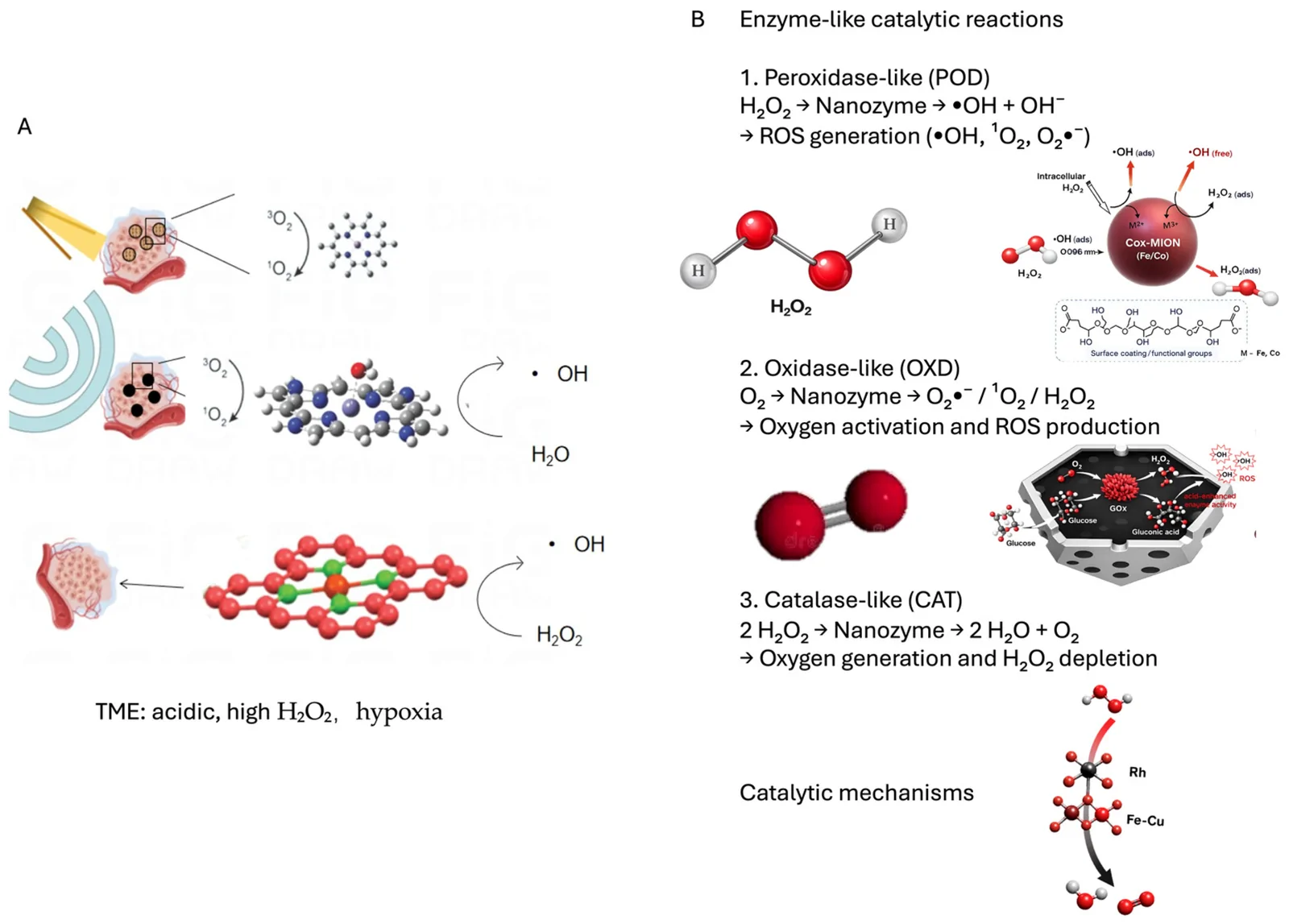
Representative catalytic and ROS-generating mechanisms of nanozymes. (**A**) Different activation strategies by external stimulation. (**B**) Enzyme-like catalytic activities of nanozymes.

Chemodynamic therapy (CDT) is a representative example of catalytic activation and encompasses several catalytic strategies that exploit tumor microenvironment conditions to generate therapeutic effects. One example involves TME-responsive nanoreactors containing Fe_3_O_4_ nanoparticles and GOx, which consume intratumoral glucose and generate gluconic acid and H_2_O_2_, enhancing local acidity and supplying substrates for the Fenton reactions (Figure 4A) [223]. Other CDT-related mechanisms include peroxidase-like (POD) activity, which converts H_2_O_2_ into hydroxyl radicals (•OH) and other ROS [224]; oxidase-like (OXD) activity, which promotes oxygen activation and ROS generation, in a process involving superoxide anions (O_2_•^−^), singlet oxygen (^1^O_2_), and H_2_O_2_ [225]; and catalase-like (CAT) activity, which decomposes H_2_O_2_ into H_2_O and O_2_, alleviating hypoxia and modulating oxidative stress (Figure 4B) [226]. Together, these catalytic processes may contribute to ROS-mediated cancer therapy.

External stimuli such as near-infrared (NIR) light, ultrasound, and electric fields can activate or enhance catalytic activity, enabling spatiotemporal control of treatment. For instance, NIR-activated nanocatalysts can consume intracellular GSH while generating hydrogen (H_2_) in a sustained and controllable manner, disrupting cancer cell metabolism and redox homeostasis, ultimately promoting DNA damage and apoptosis [227]. Compared with conventional catalysts and chemotherapeutic drugs, nanocatalysts, relying on localized and remotely activated catalytic reactions, offer greater tumor specificity and may reduce systemic toxicity. However, released Fe, Cu, or Mn ions can still induce oxidative stress in healthy tissues [228]. Therefore, future translational developments should prioritize TME-restricted metal release and improved local metal utilization to maximize therapeutic efficacy while minimizing off-target exposure of healthy tissues. Photodynamic therapy (PDT), based on photocatalytic reactions, uses light, photosensitizers, and oxygen to generate highly reactive singlet oxygen, causing tumor cell death, microvasculature damage, and local inflammation. However, its clinical application is limited by the poor penetration depth of light, making it mainly suitable for superficial tumors [229]. Sonodynamic therapy (SDT) employs ultrasound and sonosensitizers to generate reactive radicals from H_2_O and O_2_. Due to the high tissue penetration of ultrasound, SDT can effectively target deeper tissues. Its mechanism is generally associated with sonoluminescence and pyrolysis processes induced by ultrasound-mediated cavitation and thermal effects [230].

Overall, despite the promising therapeutic performance, these MNP-based systems still face challenges in terms of penetration depth, controllability, long-term biosafety, and clinical translation, highlighting the need for further optimization toward more effective, safe, and scalable nanoplatforms.

### 5.3. Evaluation and Prediction of MNP Therapeutic Performance

The therapeutic efficacy of MNPs depends on multiple factors operating across different biological scales, from molecular interactions and cellular uptake to tissue penetration and whole-body biodistribution. Consequently, a range of experimental and computational approaches has been developed to evaluate and predict MNP performance in hyperthermia, targeted drug delivery, and other therapeutic applications.

#### 5.3.1. Mechanisms and Quantitative Evaluation of Magnetic Hyperthermia

Among the various therapeutic modalities associated with MNPs, magnetic hyperthermia is one of the most readily quantifiable. Accurate quantification of heat generation is essential for optimizing treatment protocols and predicting therapeutic outcomes. In principle, hyperthermia is a process in which magnetic power dissipates, generating heat. These dissipation mechanisms include Néel relaxation, Brownian relaxation, and, for particles with hysteretic behavior, hysteresis loss.

In Néel relaxation, heat is generated through repeated reorientation of the particle magnetic moment within the magnetic core of the nanoparticle. In Brownian relaxation, energy dissipation occurs through physical rotation of the entire nanoparticle within the surrounding medium. For larger or magnetically blocked nanoparticles, hysteresis losses may also contribute to heat generation, arising from the area enclosed by the magnetization hysteresis loop. In contrast, eddy-current losses, generated by electromagnetic induction and affected by particle shape and size, are several orders of magnitude lower in comparison with hysteresis losses [231] and generally negligible at the nanoscale, and contribute minimally to the overall heating process [232]. Overall, the relative contribution of these mechanisms depends on nanoparticle properties as well as the amplitude and frequency of the applied alternating magnetic field (AMF). Magnetic heat dissipation is also based on the internal and external properties of each type of MNPs and saturation temperature, also known as thermotherapy temperature. Anisotropy, intra- and inter-particle dipole interactions, ligand length, and AMF parameters all influence the collective magnetic behavior and heat-generation efficiency of MNPs. Longer ligands may increase interparticle spacing and weaken dipole–dipole interactions, whereas aggregation can strengthen magnetic coupling and alter relaxation behavior. In biological environments, protein adsorption and cellular internalization may further affect nanoparticle aggregation, rotational freedom, and heat dissipation efficiency [233].

The magnetocaloric properties of MNPs are usually characterized by the specific absorption rate (SAR) or intrinsic loss parameter (ILP) metric. Both of them describe the energy absorbed/evolved per unit mass under the influence of an AC magnetic field. SAR represents the amount of electromagnetic energy absorbed by a unit mass of matter (in this case per kg of human tissue) per unit time. It can be calculated by

$$\mathrm{SAR}=\frac{C\times \Delta T}{\Delta t}$$

where C is the specific heat capacity of the medium, *m_MNP_* is the mass of MNP and ΔT is the temperature change, over a time interval Δt [234]. SAR can also be used to express per unit mass of magnetic material (W g^−1^ MNPs). It can be calculated from the initial temperature rise of the suspension as:

$$\mathrm{SAR}=\frac{C}{m_{\mathrm{MNP}}}\frac{\Delta T}{\Delta t}$$

where m_MNP_ is the mass of MNPs. ILP was proposed to compare heating efficiency under different experimental conditions of amplitude and frequency of the magnetic field. It can be calculated by

$$\mathrm{ILP}=\frac{\mathrm{SAR}}{fH^{2}},$$

ILP normalizes SAR values according to the applied magnetic field amplitude (H) and frequency (f), enabling comparison among studies performed under different experimental conditions. In order to safely apply localized heating to patients and avoid adverse effects on healthy tissue due to electromagnetic exposure, the mentioned factors should not exceed the experimentally estimated threshold. To maximize heating efficiency, considerable effort has been devoted to the development of magnetic materials with improved SAR and ILP values, including lanthanum manganites, mixed ferrites, and core–shell nanostructures. Their performance can be tailored through modulation of particle composition, size, shape, and magnetic anisotropy. However, maximizing heating efficiency must be balanced against considerations related to biocompatibility, colloidal stability, and clinical translatability. To better estimate electromagnetic exposure, computational simulation has also been applied to analyze MNP influence on heat distribution and temperature changes in both cancerous and healthy tissues [235]. Computational modeling is emerging as a valuable, animal-free tool for optimizing treatment planning, predicting temperature distributions, and improving the safety and efficacy of magnetic hyperthermia protocols.

#### 5.3.2. Evaluation of MNP Molecular Targeting and Cellular Uptake

Comprehensive evaluation of MNP-mediated molecular targeting requires consideration of the entire targeting cascade, from receptor selection and ligand binding to cellular internalization and intracellular accumulation.

Molecular targeting is defined as the ability of ligand-functionalized MNPs to selectively recognize and bind to cancer cells through interactions with cell-surface receptors. Consequently, identification of receptors with high tumor specificity, favorable abundance, and sufficient accessibility is essential for effective targeting. Since molecular targeting relies on specific interactions between surface-functionalized MNPs and tumor-associated receptors, the identification of differentially expressed proteins (DEPs) and genes is an important first step in the selection of suitable receptors [236]. RNA sequencing and proteomic analysis enable high-throughput profiling of gene and protein expression and facilitate the identification of differentially expressed genes between tumor and normal tissues. To quantify these differences, the parameter fold change (FC) is commonly employed. In general, FC is calculated as the ratio of gene expression in tumor tissue to that in matched normal tissue, as

FC=ExpressionTumorExpression Normal

Ref. [237]. For molecular targeting, genes encoding cell-surface receptors with high fold changes are of particular interest because their selective overexpression may improve tumor specificity and the recognition efficiency of ligand-functionalized MNPs. After gene expression, protein abundances can vary by several orders of magnitude in proteomic datasets. Direct comparison of raw expression values across proteins therefore is challenging and standardization of protein abundance is necessary. One widely used approach is the Z-score transformation, which standardizes protein abundance according to the equation:

$$z=\frac{x-\mu }{\sigma },$$

where x is the abundance of a given protein in a single datapoint, μ is the mean abundance of that protein across all datasets (samples or matched controls), and σ is the corresponding standard deviation. Z-score transformation rescales each protein abundance profile to a mean of 0 and a standard deviation of 1, allowing the identification and comparison of proteins that are relatively overexpressed or underexpressed across samples by representing how many standard deviations a data point is from the mean [238]. While trying to verify the relative abundance of target receptors through different data sets, Z-score is a powerful tool and can overcome the variability between datasets [239].

Once suitable receptors have been identified and validated, nanoparticle design can be tailored to exploit these molecular targets. This process generally involves nanoparticle synthesis, surface coating and functionalization, drug loading, and conjugation of targeting ligands to the nanoparticle surface. To assess if surface functionalization has been successful, ligand conjugation efficiency is evaluated. Ligand loading quantifies the amount of targeting ligand successfully immobilized on the nanoparticle surface relative to the total amount initially introduced during fabrication. Using the correct binding density is essential to reach the highest ligand conjugation efficiency. For example, to calculate Tf (transferrin) loading density (mg Tf/g NP), the binding density *ρ* is calculated by

$$\rho =\frac{N_{\mathrm{Tf}}}{A_{\mathrm{NP}}}$$

in which *N*_Tf_ represents the number of Tf molecules bound to nanoparticles and *A*_NP_ is the total surface area (nm^2^) of all nanoparticles. For instance, with benzoboroxole-modified core–shell MNPs (MSP-AOPB NPs) as a drug carrier, the maximum binding capacity was 184 mg Tf/g with a well-controlled targeting ligand density on the surface. In vitro studies demonstrated that, by varying ligand density, excellent Tf-mediated targeting ability and enhanced cellular uptake efficacy can be obtained. The optimal ligand binding amount for achieving the highest cellular uptake efficiency was 94 mg Tf/g, which corresponds to a ligand binding density of about 0.05 Tf/nm^2^, and the binding efficiency of conjugation was higher than 90% [240]. Besides the density, the orientation of the conjugated ligands can also influence receptor recognition and targeting efficacy. MNPs have the natural advantage of being able to simultaneously interact with multiple receptors. However, to reach efficient receptor targeting, the receptor-binding epitopes need to have outward exposure and adequate spacing between adjacent ligands. Conversely, random ligand orientation can bury ligands within the PEG layer or adjacent ligands may sterically hinder one another, ultimately impairing efficient multivalent binding and reducing targeting efficacy.

Following receptor binding, cellular uptake and intracellular distribution must be evaluated to determine whether targeted MNPs effectively reach their intended cellular compartments. To evaluate whether MNP can successfully get into target cells, there are different options (Table 2). Microscopy is one of the most commonly used methods. Transmission electron microscopy (TEM) [241] provides direct visualization of MNPs within the cells and organelles, while fluorescently labeled MNPs allow for measuring the signal with fluorescent microscopes or confocal laser scanning microscopy [242]. Another method includes measurement of the amount (weight) of iron present in the target cells. The iron content per cell (MFe) is calculated by

$$M_{\mathrm{Fe}}=\frac{m_{\mathrm{Fe},\mathrm{total}}}{N_{\mathrm{cells}}}$$

where *m_Fe_*_,*total*_ represents the total iron mass (pg) in the cells, while *N_cells_* denotes the number of cells. These methods allow measurement of the quantity of MNPs within a single cell [243]. Measuring the elemental composition of cells can also quantify metal ions released from MNPs. Inductively coupled plasma mass spectrometry (ICP-MS) [244] or mass spectrometry (MS) itself can be extremely sensitive and highly accurate methods for quantifying and detecting trace amounts of iron [245]. In flow cytometry, the degree of uptake is assessed by utilizing changes in the intensity of laser scattering by cells. Forward scattering (FSC) primarily reflects cell size, while side scattering (SSC) is commonly used as an indirect indicator of intracellular complexity and granularity.

MNP internalization leads to increased intracellular granularity and higher relative SSC (RSS). This can be calculated by using the following formula:

$$\mathrm{RSS}=\frac{\mathrm{SS}C_{\mathrm{cells}+\mathrm{MNPs}}}{\mathrm{SS}C_{\mathrm{control cells}}}$$

where SSC_cells+MNPs_ is the SSC of cells after MNP uptake and SSC_control cells_ is the SSC of untreated cells. For instance, due to particle endocytosis, the median RSS of cells can significantly increase from an initial 142 to 806, while FSC, proportional to cell size, remains essentially unchanged [246]. The above-mentioned methods all have different advantages and disadvantages (Table 2). Other methods include MPI [247] and surface-enhanced Raman spectroscopy (SERS) [248]. These focus on magnetic response and Raman signals from the target cells. However, both techniques require specialized instrumentation and remain less accessible and more challenging than conventional microscopy- or cytometry-based approaches.

In addition to quantifying the extent of cellular uptake, it is also important to evaluate the kinetics and intracellular distribution of MNP internalization. One useful parameter is the partitioning ratio (ratio between adhesion and uptake), which describes the distribution of nanoparticles between the cell surface and intracellular compartments. It is calculated as the proportion of cell-associated nanoparticles that are successfully internalized following adhesion to the plasma membrane. As such, partitioning provides an indication of the efficiency with which surface-bound nanoparticles enter the cell. Prior to internalization, nanoparticles interact with the cell surface, and their physicochemical properties largely determine the endocytic pathways involved [249]. Beyond the extent of uptake, the rate of nanoparticle internalization can also be quantified. The ingestion kinetic constant (τ) describes the dynamics of endocytic uptake as a function of time. Experimentally, τ for endocytosis has been estimated to be approximately 60 ± 10 min. Accordingly, nanoparticle uptake is frequently modeled using first-order kinetics [250], where M(t) is the cellular iron content at time t (pg Fe/cell), Mmax is the maximum uptake at equilibrium, t is the incubation time, and τ is the endocytosis kinetic constant (h or min). Ligand modification and cell type are essential factors in optimizing cellular uptake efficiency. For example, by altering the ligand density, the uptake of Tf-MSP-AOPB NPs in HepG2 cells was 25 times and 127 times that of physical adsorption and EDC/NHS methods, respectively. These results indicate that transferrin-mediated targeting significantly enhanced both cellular uptake efficiency and overall targeting performance [240].

On a systemic scale, to reach the target tumor cell, we also need to ensure a certain level of physicochemical stability. This can be assessed by quantifying the MNP aggregation state in the blood vessels. The physical state of MNPs in biological environments critically influences both their safety and therapeutic performance. The hydrodynamic diameter (DH) is a sensitive parameter for assessing nanoparticle aggregation and colloidal destabilization in protein-rich media. Particle aggregation is strongly influenced by the composition of the surrounding physiological medium. For instance, unstable citrate-coated particles rapidly aggregate from 19 nm to approximately 3 µm clusters in RPMI medium, while PAA-coated particles remain stable at approximately 28 nm. Mathematical models have shown that external magnetic fields may enhance drug penetration into solid tumors by increasing the local retention of drug-loaded MNPs and promoting their diffusion from tumor-associated vessels into the tumor tissue [251]. Vascularized organ models, such as lung cancer models, have also been applied to mimic more complicated in vivo biological environments [252].

#### 5.3.3. Clinical Translation of MNP-Based Cancer Therapies: Current Status, Key Challenges, and Future Perspectives

Despite extensive preclinical development, clinically approved MNP systems for cancer treatment remain very limited. NanoTherm^®^ represents the main clinically translated platform for direct magnetic nanoparticle-mediated cancer therapy [253], whereas Magtrace^®^/Sentimag^®^ is approved for magnetic sentinel lymph node localization in breast cancer rather than direct tumor treatment [254]. Representative examples of clinical and regulatory applications are summarized in Appendix A, while selected animal studies are presented in Appendix B.

Several factors contribute to the limited clinical translation of magnetic nanoparticle formulations. From a therapeutic perspective, the first major challenge is the difficulty of controlling in vivo biodistribution. Animal experiments often show good tumor enrichment, but human tumors are more heterogeneous. Many MNPs ultimately enter the liver, spleen [255], and mononuclear phagocyte system [256], with an unstable proportion actually reaching cancer cells. Magnetothermal therapy requires simultaneously ensuring sufficient particle quantity and uniform distribution within the tumor [257], while the applied magnetic field strength and frequency must not exceed safe limits. For inoperable tumors with deep location, large volume, and irregular shape, it is difficult to heat only the tumor without damaging normal tissue [258]. Secondly, long-term safety and clearance in the patient’s body are complex issues. Iron oxides are relatively biocompatible, but coating, particle size, surface ligands, and degradation products can all be associated with inflammation, oxidative stress, immune responses, and long-term accumulation [259]. Regulatory requirements go beyond short-term non-toxicity. Batch consistency, long-term toxicity, metabolism, and excretion must all be clearly defined. Taken together, these challenges may limit the perceived clinical advantages offered by MNP-based approaches. Regulatory agencies, healthcare providers, and reimbursement systems ultimately require clear evidence that such approaches offer meaningful benefits over established treatment modalities, including radiotherapy, chemotherapy, surgery, ablation techniques, and existing nanomedicine-based approaches. Improvements in tumor size control observed in preclinical animal models alone are unlikely to justify costly late-stage clinical development unless they translate into enhanced survival, reduced toxicity, or therapeutic options for otherwise inoperable tumors.

Beyond these therapeutic and safety-related limitations, the translation of MNP-based therapies is further complicated by manufacturing and quality-control challenges. The biological behavior of MNPs is highly dependent on their physicochemical properties, which are easily affected by manufacturing processes. From a preparation and handling perspective, quality control during manufacturing is very difficult. Nanomedicines are extremely sensitive to particle size, PDI, surface charge, ligand density, and magnetic parameters. A batch that performs well in the lab does not guarantee that every batch will be the same after industrial scale-up [260]. MNPs intended for intravenous, intratumoral, or interstitial injection must meet sterility requirements, but the sterilization process itself can alter the nanoparticle structure. Multifunctional MNPs present additional challenges. Incorporating imaging capabilities, therapeutic cargoes, targeting ligands, and ROS-responsive moieties within a single platform increases system complexity and consequently raises both CMC and regulatory requirements. Commercialization and equipment barriers are also high. NanoTherm is not just a drug; it also requires specialized AMF equipment, treatment planning, local injection procedures, and training. In other words, it is a drug–device combination product, making hospital deployment costs and regulatory complexity higher than with regular intravenous medications. Storage stability is also an issue. Long-term storage of MNP suspensions must avoid aggregation, sedimentation, coating degradation, oxidation, and deterioration of magnetic properties [261], as these variations can lead to a final formulation for patients that differs from the initial development.

As a matter of fact, although NanoTherm^®^ obtained CE marking in Europe, its clinical implementation has remained largely restricted to Germany, primarily for recurrent or inoperable brain tumors [262]. Other EU countries have not adopted the therapy, and regulatory approval by the FDA in the United States has not been achieved, although an IDE (Investigational Device Exemption) has been granted for experimental clinical studies. So far the latter has been applied to a single study on the focal ablation of intermediate-risk prostate cancer with the NanoTherm therapy, which was, however, never completed (NCT05010759). Limited clinical efficacy, high infrastructural requirements, and the subsequent insolvency of NanoTherm have further prevented broader international implementation, although MagForce did announce additional supportive data from stage 2a of its pivotal U.S. single-arm study prior to its insolvency [263].

Similarly, ferumoxytol-based products have mainly received regulatory approval for diagnostic imaging or iron replacement therapy rather than cancer treatment, highlighting the gap that still exists between experimental MNP applications and routine oncological practice. The regulatory status of ferumoxytol depends on the specific product and indication. Feraheme^®^ (ferumoxytol injection), a carbohydrate-coated superparamagnetic iron oxide formulation, was initially approved by the FDA in 2009 as an intravenous iron-replacement product for iron-deficiency anemia in adults who cannot tolerate or have responded inadequately to oral iron, or who have chronic kidney disease. Feraheme is not approved as an anticancer therapy, and its use as an MRI contrast agent in earlier oncology studies was investigational or off-label. In October 2025, a separate ferumoxytol product, Ferabright™, received FDA approval for diagnostic imaging as an iron-based contrast agent for brain MRI in adults with known or suspected malignant brain neoplasms, specifically to visualize lesions with a disrupted blood–brain barrier, but not for cancer treatment. Ferumoxytol products also carry warnings regarding potential serious hypersensitivity reactions, hypotension, iron overload, and interference with subsequent MRI examinations [264,265].

#### 5.3.4. Long-Term Safety and Toxicity of MNPs

Long-term toxicity is a central issue for the clinical translation of MNP-based therapies. Key determinants include biodegradation, iron metabolism, organ accumulation, immune responses, clearance pathways, nanoparticle aggregation, protein corona formation, and the potential toxicity of surface coatings or doped metallic components. Because the liver and spleen exhibit high retention and phagocytic activity, MNPs accumulate predominantly in these organs [266]. Iron oxide MNPs are mainly internalized by macrophages and degraded within lysosomes, and interactions between apoferritin and MONPs in the liver, spleen, and inflammatory macrophages further contribute to their breakdown [267].

Experimental evidence suggests that iron status may strongly influence MNP degradation kinetics [268]. Under iron-deficient conditions, MNP degradation accelerates, and released iron is rapidly incorporated into hemoglobin. Under iron-sufficient conditions, the spleen maintains high iron levels, while the liver gradually metabolizes its degradation products. Due to their distinct physiological roles, the spleen—responsible for iron recycling—degrades MNPs more rapidly, whereas the liver, functioning as an iron storage organ, degrades them more slowly. MNP degradation is also dose-dependent: higher doses slow degradation and reduce iron utilization in iron-deficient states. Interestingly, compared with intravenous iron sucrose, MNPs have been reported to exhibit higher bioavailability and more efficient iron supplementation [268].

Clearance profiles vary among formulations: most MNPs exhibit biexponential clearance, while some follow monoexponential kinetics [269]. Intact MNPs are rarely eliminated efficiently by the kidneys, although renal clearance remains possible for certain inorganic nanoparticles depending on their surface chemistry, size, and shape [270].

MNPs can also activate the complement component C3, leading to cytokine release, opsonization, endothelial activation, and enhanced monocyte/phagocyte recognition, resulting in increased clearance [271]. However, this response shows substantial interpersonal variability, with complement activation differing by up to 26-fold among individuals exposed to the same MNP formulation [272].

Given these complexities, well-defined pharmacokinetics, safety profiles, and regulatory standards are essential. Collaboration between academia and regulatory agencies will be critical for establishing unified criteria that support clinical translation and commercialization of MNP-based therapies. Although released iron can enter normal metabolic pathways, excessive accumulation may promote oxidative stress, chronic inflammation, and tissue injury through iron-mediated ROS generation. Standardized toxicity assessment should therefore include particle characterization in biological media, endotoxin and blood-compatibility testing, complement and oxidative-stress assays, repeated-dose biodistribution, liver and kidney function, organ histology, and sufficiently long follow-up. Further long-term preclinical and clinical investigations will be required to establish standardized safety criteria and better define the long-term risk profile of MNP-based therapies.

## 6. Conclusions and Future Perspectives

Over the past decade, emerging platforms such as organ-on-a-chip models and AI-based diagnostic and predictive technologies have shown considerable promise towards the development of personalized medicine. Yet, despite these advances, conventional treatment modalities, including chemotherapy, radiotherapy, and surgery, remain the backbone of clinical oncology. Within this evolving landscape, MNPs have received increasing recognition because of their ability to interface with both established and novel therapeutic strategies. The advantage of MNPs lies in their capacity to be remotely controlled and directed to the tumor site, where they can induce targeted hyperthermia, drug release, and be part of combination therapies triggering various cancer cell death mechanisms. These characteristics potentially enable their application across multiple stages of cancer management. To further enhance their performance, current design efforts are increasingly directed toward tumor-specific hallmarks, such as acidic pH, ATP overproduction, altered glucose metabolism, and elevated stiffness, alongside systemic features like immune cell dynamics and vascular abnormalities. This shift is giving rise to more adaptive, stimuli-responsive nanoplatforms that hold promise for smarter and less toxic treatment side effects.

However, while MNP-based approaches have shown promising results for blood cancers such as leukemia and lymphoma, their application in solid tumors is still at the very beginning. Importantly, these limitations are not confined to a single scale. At the nanoscale, challenges persist in material design, including poor physicochemical properties, batch-to-batch variability, and low production efficiency. At the cellular and molecular levels, variable uptake mechanisms, differential cytotoxic responses, and lysosomal degradation of therapeutic payloads contribute to heterogeneous outcomes. At the tissue and organism levels, non-uniform EPR effects, dense and compositionally diverse extracellular matrices, and dynamically evolving microenvironments inhibit adequate MNP penetration and biodistribution. Clinically, off-target accumulation in vascular-rich organs, and clearance time, safety, and scalability concerns remain key barriers.

Taken together, these multiscale barriers underscore that no single solution will be enough to address all these challenges. Future research must therefore combine advanced materials engineering, precise control over cellular interactions, microenvironment-responsive systems, and improved in vivo delivery and monitoring strategies. Rather than relying on a one-size-fits-all formulation, future MNP platforms should be tailored according to patient- and tumor-specific characteristics. Such an approach could support precision oncology by guiding the selection of targeting ligands, therapeutic cargoes, administration routes, MNP doses, and magnetic field parameters for individual patients. Equally important is the need for closer collaboration among academic laboratories, industry partners, and regulatory bodies, which will be essential for establishing standardized evaluation frameworks and accelerating the safe and effective translation of MNP-based technologies into routine clinical practice.

## Figures and Tables

**Table 2:** Overview and comparison of commonly used evaluation methods for MNPs cellular uptake.

Method	Advantages	Limitations
Fluorescence labeling	1. Allows direct visualization of the intracellular and subcellular localization of MNPs 2. Enables studies of intracellular trafficking and distribution pathways 3. Allows real-time and dynamic tracking of MNPs.	1. Fluorescent dyes may alter the surface properties and uptake behavior of MNPs 2. Fluorescence quenching and photobleaching may occur 3. Fluorescence intensity is not always linearly correlated with particle number, thereby suggesting limiting quantitative accuracy.
Iron quantification	1. Provides accurate quantitative information on cellular uptake 2. High sensitivity 3. Relatively simple, does not require labeling.	1. Cannot provide information on the dynamics of intracellular localization 2. Usually requires cell lysis and therefore cannot be used for real-time monitoring 3. Cannot distinguish between iron bound to the cell surface and iron that has been internalized.
Flow cytometry (SSC/RSS analysis)	1. Rapid analysis of large numbers of cells 2. High-throughput method with reliable statistical analysis 3. Can reveal heterogeneity in nanoparticle uptake among cell populations 4. Can be combined with cell surface markers for multiparametric analysis.	1. Changes in SSC can also be affected by other cellular processes, such as activation or apoptosis thereby are not specific to MNP uptake 2. Cannot provide precise intracellular localization information (unless imaging Flow Cytometry is used) 3. quantifying the exact amount of MNPs internalized by individual cells is also difficult

## Data Availability

No new data were created or analyzed in this study. Data sharing is not applicable to this article.

## Abbreviations

The following abbreviations are used in this manuscript:

MNPs	Magnetic nanoparticles
TME	Tumor microenvironment
FDA	Food and Drug Administration
MRI	Magnetic resonance imaging
SLP	Specific loss power
AMFs	Alternating magnetic fields
MACS	Magnetic activated cell sorting
CTCs	Circulating tumor cells
SPIONs	Superparamagnetic iron oxide nanoparticles
HPLC	High-performance liquid chromatography
PEG	Polyethylene glycol
PVA	Polyvinyl alcohol
2D	Two-dimensional
3D	Three-dimensional
AI	Artificial intelligence
TfR1	Transferrin receptor 1
SCLC	Small cell lung cancer
LHRH	Luteinizing hormone-releasing hormone
RME	Receptor-mediated endocytosis
PAA	Polyacrylic acid
CIE	Clathrin-independent endocytosis
FEME	Fast endophilin-mediated endocytosis
CSCs	Cancer stem cells
ROS	Reactive oxygen species
IFP	Interstitial hydraulic pressure
EPR	Enhanced permeability and retention
GSH	Glutathione
RES	Reticuloendothelial system
BBB	Blood–brain barrier
MPI	Magnetic particle imaging
MDR	Multidrug resistance
TAMs	Tumor-associated macrophages
RMF	Rotating magnetic field
GOx	Glucose oxidase
APC	Antigen-presenting cell
IP	Intraperitoneal
EGF	Epidermal growth factor
TH	Thermotherapy temperature
SAR	Specific absorption rate
ILP	Intrinsic loss parameter
ECM	Extracellular matrix
PLGA	Poly(lactic-co-glycolic acid)
PHEUU	Polyurethane urea
PDT	Photocatalytic therapy
SDT	Sonocatalytic therapy
CDT	Chemodynamic therapy
POD	Peroxidase-like activity
OXD	Oxidase-like activity
CAT	Catalase-like activity
MMP	Matrix metalloproteinase
STING	Stimulator of interferon genes
NIR	Near-infrared
DEPs	Differentially expressed proteins
FC	Fold change
SSC	Side scattering
FSC	Forward scattering
RSS	Relative side scatter
MS	Mass spectrometry
SERS	Surface-enhanced Raman spectroscopy

## Appendix A

To complement the discussion of the biological behavior and therapeutic potential of MNPs, this appendix summarizes representative MNP-based platforms that have progressed to clinical investigation or animal evaluation in oncology. Table A1 presents their nanoparticle formulations or commercial products, clinical indications, therapeutic or diagnostic mechanisms, principal efficacy and safety findings, and regulatory status. Therapeutic, imaging, and surgical-localization applications are distinguished; authorization for imaging or localization does not imply approval for cancer treatment. Collectively, these examples illustrate both the translational potential of MNPs and the remaining challenges associated with clinical efficacy, treatment standardization, patient selection, large-scale manufacturing, and regulatory approval. Because the clinical and regulatory status of these platforms may evolve, the information presented reflects the evidence available at the time of the literature search.

**Table A1:** Clinical translation of magnetic nanoparticle-based platforms in oncology.

Nanoparticle Formulation/Product	Clinical Indication	Principal Efficacy and Safety Findings	Regulatory Status	Reference
NanoTherm^®^, aminosilane-coated SPIONs Prospective, single-arm phase II study	Recurrent glioblastoma; local treatment combined with reduced-dose radiotherapy 66 treated; 59 with recurrent GBM; neuronavigational intratumoral injection	Median OS after first recurrence was 13.4 months and median OS from initial diagnosis was 23.2 months; adverse effects were reported as moderate, without serious treatment-related complications; interpretation is limited by the single-arm design and historical comparison	Received European CE marking in 2010 for use in brain tumors; no FDA marketing authorization in the United States	[273]
NanoTherm-related magnetic fluid hyperthermia Prospective phase I feasibility study	Locally recurrent prostate cancer; feasibility of local prostate thermotherapy 10 patients; transperineal intraprostatic injection	Intraprostatic temperatures up to 55 °C were achieved; nanoparticle deposits could be detected by CT; the study primarily established technical feasibility rather than oncological efficacy	Investigational use; not FDA-approved for prostate cancer	[274]
NanoTherm^®^ Therapy System Prospective, single-arm, device study; phase not applicable	Limited-volume, localized intermediate-risk prostate cancer; focal tumor ablation 3 patients; intralesional/intraprostatic instillation	No results were posted; the study was terminated because of inadequate enrollment and a change in company direction	FDA-regulated investigational device; unapproved/uncleared in the United States	NCT05010759
NanoTherm^®^ Therapy System Interventional study; phase not applicable	Adjuvant treatment after surgery, with standard oncological treatment; planned: 100; application to the tumor/resection region	Recruitment/assessment ongoing; no definitive efficacy or safety results available	Investigational study in Poland; European clinical investigation	NCT06271421
Magtrace^®^/Sentimag^®^; carboxydextran-coated superparamagnetic iron oxide tracer and magnetic probe Prospective, multicenter, paired noninferiority pivotal trial	Breast cancer/DCIS; sentinel lymph-node localization during breast surgery 147 patients; evaluable; interstitial, subareolar, or peritumoral injection according to the approved procedure	Per-patient detection was 99.3% with Magtrace versus 98.6% with radioisotope plus blue dye; per-node detection was 94.3% versus 93.5%	FDA PMA approval in 2018 for breast-cancer sentinel-node localization; indication subsequently expanded. It is a surgical localization system, not a direct anticancer therapy	NCT02336737; FDA PMA P160053
SPIONs with a handheld magnetic probe Prospective multicenter clinical trial	Clinically node-negative breast cancer; sentinel lymph-node biopsy 220 patients; subcutaneous/subareolar injection	Demonstrated high sentinel-node identification performance; intended as a radioisotope-free localization method	Clinical investigation; regulatory status depends on the specific tracer and jurisdiction	[275]
FerroTrace^®^, mannose-labeled iron oxide nanoparticles Phase I, first-in-human prospective study	N0 oral squamous cell carcinoma; preoperative and intraoperative sentinel lymph-node localization 8 patients; peritumoral injection	All 21 MRI-identified sentinel nodes were located intraoperatively; the only metastatic node was identified, and no adverse events were reported; diagnostic accuracy cannot be established from this small cohort	Investigational; not an approved anticancer therapy	[276]
FerroTrace^®^/FerroMag system Feasibility clinical investigation	Gastric and gastroesophageal cancer; magnetic sentinel lymph-node mapping	Clinical outcome data remain unavailable or preliminary	Investigational	NCT05899985
FerroTrace^®^ with indocyanine green Phase I diagnostic study	Gastric cancer; sentinel lymph-node imaging and mapping during gastrectomy	Remain unavailable	Investigational	NCT05038098
SN132D/pegfosimer manganese (SpagoPix), a PEGylated polymeric nanoparticle containing chelated Mn^2+^ Phase I, first-in-human, open-label study	Breast cancer; contrast-enhanced MRI of primary tumors 14 patients in the pharmacokinetic analysis; intravenous infusion	Pharmacokinetics were consistent with size-dependent renal filtration; the model fit was R^2^ = 0.991, with an estimated filtration cutoff of approximately 5.5 nm and little or no plasma-protein binding; this publication did not establish therapeutic efficacy	Investigational imaging agent; not an approved anticancer therapy	NCT04080024
Ferumoxytol, marketed as Feraheme^®^ for iron replacement Phase II, open-label diagnostic study	Experimental MRI assessment of tumor vascularity and permeability 40 started; 33 completed	Supported assessment of tumor vascularity and comparison with gadolinium-enhanced MRI; the study was diagnostic and did not evaluate antitumor efficacy	Feraheme is FDA-approved for iron-deficiency anemia, not cancer treatment. Its use as an MRI agent in this study was investigational/off-label	NCT00659126
Ferabright™ (ferumoxytol injection) FDA-reviewed diagnostic development program	Known or suspected malignant brain neoplasms; MRI visualization of lesions with disruption of the blood–brain barrier; intravenous infusion	Approved for MRI visualization rather than treatment; carries warnings concerning serious hypersensitivity reactions, hypotension, iron overload, and effects on subsequent MRI examinations	FDA-approved in October 2025 for brain MRI in adults with known or suspected malignant brain neoplasms; not approved as an anticancer therapy	FDA NDA 219868

## Appendix B

This appendix provides a summary of representative animal studies evaluating MNP-based therapeutic strategies in oncology. As shown in Table A2, these studies encompass magnetic hyperthermia, photothermal therapy, chemotherapy, ferroptosis induction, and immune modulation across different tumor models. The available findings suggest the potential of MNP-based platforms to inhibit primary tumor growth and metastatic progression in the models examined. However, differences in nanoparticle formulation, animal model, administration route, treatment schedule, and outcome assessment limit direct comparisons among studies. Additional concerns, including heterogeneous intratumoral distribution, off-target organ retention, treatment complexity, and limited long-term safety data, should also be addressed before these approaches can progress toward clinical translation.

**Table A2:** Selected examples of animal studies evaluating MNP-based therapeutic strategies in oncology.

Therapy	Treatment Model and Conditions	Main Therapeutic Outcome	Key Limitations	Ref.
Chemo–photothermal combination	DOX-loaded liposomal IONPs; B16-F10 melanoma-bearing mice DOX 4 mg/kg and IONPs 24 mg Fe/kg; magnetic targeting and PTT; three treatments on Days 9, 11, and 13 after tumor implantation	84% tumor-growth inhibition, exceeding free DOX and non-irradiated formulations	Complex formulation and treatment schedule; requires both magnetic targeting and light exposure	[277]
Chemo–ferroptosis combination	DOX-loaded liposomal iron oxide 4T1 tumor-bearing Balb/c mice DOX 2.5 mg/kg and Fe 1 mg/kg, administered daily for 7 days	63.9% tumor-volume inhibition versus 36.3% for DOX and 30.8% for Lp–IO alone	Potential cumulative toxicity and lack of long-term relapse assessment	[278]
Immune modulation/metastasis inhibition	Iron oxide “batch-p” nanoparticles; human HER2-overexpressing transgenic mice (HuHER2); single intravenous injection of 5 mg Fe/mouse	Lung metastases occurred in 0/10 treated mice versus 5/12 controls at endpoint	Antitumor activity was formulation-specific and accompanied by high liver and spleen retention	[279]
Hyperthermia alone	Prostate tumors in rats; intratumoral injection of SPIONs followed by repeated exposure to an alternating magnetic field	Tumor growth was inhibited by 44–51% compared with controls	Heterogeneous intratumoral particle distribution; optimal treatment temperature and schedule remained undefined	[280]
